# Mutations in tropomyosin 4 underlie a rare form of human macrothrombocytopenia

**DOI:** 10.1172/JCI86154

**Published:** 2017-01-30

**Authors:** Irina Pleines, Joanne Woods, Stephane Chappaz, Verity Kew, Nicola Foad, José Ballester-Beltrán, Katja Aurbach, Chiara Lincetto, Rachael M. Lane, Galina Schevzov, Warren S. Alexander, Douglas J. Hilton, William J. Astle, Kate Downes, Paquita Nurden, Sarah K. Westbury, Andrew D. Mumford, Samya G. Obaji, Peter W. Collins, NIHR BioResource, Fabien Delerue, Lars M. Ittner, Nicole S. Bryce, Mira Holliday, Christine A. Lucas, Edna C. Hardeman, Willem H. Ouwehand, Peter W. Gunning, Ernest Turro, Marloes R. Tijssen, Benjamin T. Kile

**Affiliations:** 1 Walter and Eliza Hall Institute of Medical Research, Parkville, Australia.; 2 Department of Medical Biology, University of Melbourne, Parkville, Australia.; 3 Department of Haematology, University of Cambridge, Cambridge Biomedical Campus, Cambridge, United Kingdom.; 4 NHS Blood and Transplant, Cambridge Biomedical Campus, Cambridge, United Kingdom.; 5 Institute of Experimental Biomedicine, University Hospital and Rudolf Virchow Center, University of Wuerzburg, Wuerzburg, Germany.; 6 School of Medical Sciences, University of New South Wales, Sydney, Australia.; 7 Institut Hospitalo-Universitaire LIRYC, Plateforme Technologique d’Innovation Biomédicale, Hôpital Xavier Arnozan, Pessac, France.; 8 School of Clinical Sciences, University of Bristol, Bristol, United Kingdom.; 9 School of Cellular and Molecular Medicine, University of Bristol, Bristol, United Kingdom.; 10 Arthur Bloom Haemophilia Centre, School of Medicine, Cardiff University, Heath Park, Cardiff, United Kingdom.; 11 NIHR BioResource–Rare Diseases, Cambridge University Hospitals, Cambridge Biomedical Campus, Cambridge, United Kingdom.; 12 Transgenic Animal Unit, Mark Wainwright Analytical Centre, University of New South Wales, Sydney, Australia.; 13 Human Genetics, Wellcome Trust Sanger Institute, Wellcome Trust Genome Campus, Hinxton, United Kingdom.; 14 Medical Research Council Biostatistics Unit, Cambridge Institute of Public Health, Cambridge, United Kingdom.

## Abstract

Platelets are anuclear cells that are essential for blood clotting. They are produced by large polyploid precursor cells called megakaryocytes. Previous genome-wide association studies in nearly 70,000 individuals indicated that single nucleotide variants (SNVs) in the gene encoding the actin cytoskeletal regulator tropomyosin 4 (TPM4) exert an effect on the count and volume of platelets. Platelet number and volume are independent risk factors for heart attack and stroke. Here, we have identified 2 unrelated families in the BRIDGE Bleeding and Platelet Disorders (BPD) collection who carry a *TPM4* variant that causes truncation of the TPM4 protein and segregates with macrothrombocytopenia, a disorder characterized by low platelet count. *N*-Ethyl-*N*-nitrosourea–induced (ENU-induced) missense mutations in *Tpm4* or targeted inactivation of the *Tpm4* locus led to gene dosage–dependent macrothrombocytopenia in mice. All other blood cell counts in *Tpm4*-deficient mice were normal. Insufficient TPM4 expression in human and mouse megakaryocytes resulted in a defect in the terminal stages of platelet production and had a mild effect on platelet function. Together, our findings demonstrate a nonredundant role for *TPM4* in platelet biogenesis in humans and mice and reveal that truncating variants in *TPM4* cause a previously undescribed dominant Mendelian platelet disorder.

## Introduction

Platelets are small, anuclear blood cells that are essential for blood clotting and the maintenance of vascular integrity (1, 2). They are produced by megakaryocytes, large polyploid precursor cells that develop primarily in the bone marrow. Megakaryocytes undergo a unique process of growth and maturation involving replication of DNA without cell division (endomitosis), a massive expansion of cytoplasmic volume, and the development of an extensive internal membranous network known as the demarcation membrane system. Upon maturity, megakaryocytes form filamentous protrusions termed “proplatelets” that are extruded into the bone marrow sinusoids. Evidence suggests platelet biogenesis is the result of individual platelets budding off from proplatelets, as well as proplatelets themselves being shed and subsequently maturing into platelets within the circulation (3). The molecular regulation of this unique process of blood cell formation is only partially understood.

The number, cellular volume, and function of platelets are tightly regulated within individuals. However, all 3 parameters vary widely at the population level, and represent independent risk factors for heart attack and stroke (4). Population variation is to a large extent explained by common single nucleotide variants (SNVs), which exert small effects. Major deviations are triggered by rare variants that have effects of sufficient magnitude to cause Mendelian platelet disorders. A metaanalysis of genome-wide association studies identified 68 independent common SNVs associated with platelet count and volume (5). Several of these SNVs were linked to genes encoding proteins with well-established roles in platelet formation (e.g., *THPO* [thrombopoietin], *GP1BA* [glycoprotein Ibα], *TUBB1* [tubulin-β1]) and survival (the apoptosis regulator *BAK* [encoding BAK]). However, the vast majority of identified SNVs were located near genes hitherto uncharacterized in the megakaryocyte lineage.

Tropomyosins, which form copolymers with actin, are a prime example, with SNVs rs3809566 and rs8109288 marking the genes encoding tropomyosin 1 (*TPM1*) and tropomyosin 4 (*TPM4*), respectively. It has been reported that expression of transcripts from the *TPM4* locus strongly increases in the later stages of megakaryopoiesis (5, 6). In addition, chromatin immunoprecipitation in primary megakaryocytes for 5 transcription factors with essential roles in platelet biogenesis revealed that *TPM4* contains a nucleosome-depleted regulatory element occupied by all 5 factors (7), placing the gene in a unique category of fewer than 200 genes. Morpholino knockdown of its homolog *tpma* in zebrafish abolished the formation of thrombocytes, suggesting a functional role for *TPM4* in mammalian thrombopoiesis (5, 8).

Tropomyosins are ubiquitously expressed and highly conserved. Mouse and human homologs exhibit greater than 90% identity at the amino acid level. In mammals, tropomyosins are encoded by 4 different genes: *TPM1*, *TPM2*, *TPM3*, and *TPM4*. Alternative promoter usage and alternative splicing result in approximately 40 different TPM protein isoforms in humans (reviewed in ref. 9; for nomenclature used in this study, see ref. 10). Intriguingly, despite their similarity, individual tropomyosin genes have largely nonredundant functions, as illustrated by the fact that the constitutive deletion of either *Tpm1*, *Tpm2*, or *Tpm3* in mice results in embryonic lethality (11–13). *Tpm4* knockout mice have not previously been described.

The best-characterized tropomyosins are the muscle-specific isoforms, which mediate the interaction between myosin and actin. In humans, mutations affecting the expression of muscle tropomyosins cause familial hypertrophic cardiomyopathy (*TPM1*) and nemaline myopathies (*TPM2* and *TPM3*). These disease phenotypes are recapitulated in mice carrying similar mutations (14). The “nonmuscle” (or “cytoskeletal”) tropomyosin isoforms are often referred to as “gatekeepers,” as they regulate the accession of other actin-binding proteins and myosin motors to the actin filament (9). The cytoskeletal tropomyosins are implicated in the regulation of cell morphology, adhesion, migration, granule trafficking, cell division, and apoptosis. Except for an involvement in modulating erythrocyte membrane stability (15), virtually nothing is known about the roles of tropomyosins in hematopoietic cells.

Here, we demonstrate that, in both humans and mice, TPM4 is an essential mediator of platelet biogenesis. Genome sequencing of 865 index cases with bleeding and platelet disorders of unknown molecular etiology revealed 2 pedigrees with a premature stop codon in *TPM4*. This variant was absent from nearly 75,000 control DNA samples and segregated with large platelets and concomitant low platelet count (a phenotype termed macrothrombocytopenia) in the 2 pedigrees. Mutation of murine *Tpm4* resulted in dose-dependent macrothrombocytopenia caused by a defect in the terminal stages of platelet production. This was recapitulated by shRNA knockdown in human megakaryocytes. In addition to a reduction in platelet number and an increase in mean platelet volume, a mild defect in platelet function was observed in both humans and mice. Our findings demonstrate that *TPM4* encodes a tropomyosin with an important nonredundant role in platelet biogenesis, and define a novel, dominant, Mendelian platelet disorder.

## Results

### Tropomyosin expression in human and mouse megakaryocytes and platelets.

**Figure 1:**
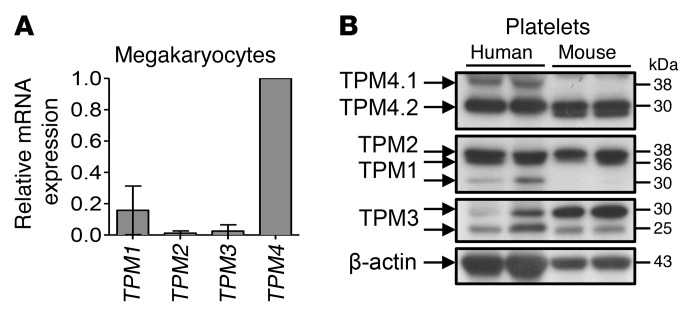
Tropomyosin expression in human and mouse. (**A**) Multiple TPMs are expressed in human megakaryocytes. Relative *TPM* mRNA expression of human cord blood–derived megakaryocytes was evaluated by real-time quantitative PCR. Data are presented as mean ± SD of 3 independent experiments. (**B**) Comparison of tropomyosin (TPM1–4) protein expression between human and WT mouse platelets. Top panel: The 30-kDa TPM4 protein isoform (TPM4.2) is the main isoform in both human and mouse platelets. Human platelets additionally express low amounts of the 38-kDa TPM4 isoform (TPM4.1). Bottom panel: β-Actin loading control.

Previous RNA sequencing studies have indicated that human megakaryocytes express *TPM1*, *TPM3*, and *TPM4*, and low levels of *TPM2* (ref. 6 and Supplemental Figure 1A; supplemental material available online with this article; https://doi.org/10.1172/JCI86154DS1). We performed reverse transcriptase–PCR (RT-PCR), which confirmed these findings, and demonstrated that *TPM4* is the most highly expressed of the tropomyosins, followed by *TPM1* (Figure 1A). Western blotting of whole cell lysates demonstrated the presence of protein products of all 4 tropomyosin genes in WT mouse and healthy human control platelets (Figure 1B). The short TPM4 30-kDa isoform (TPM4.2) was the major isoform in both species. Human platelets additionally appear to harbor small amounts of the long 38-kDa TPM4.1 isoform (Figure 1B, top). Hereafter, the term “TPM4” is used to indicate both protein isoforms TPM4.1 and TPM4.2 (in humans) or solely TPM4.2 (in mice).

### A truncating mutation in TPM4 causes macrothrombocytopenia in humans.

We next performed a look-up in the database for the Biomedical Research Centres/Units Inherited Diseases Genetic Evaluation (BRIDGE) Bleeding and Platelet Disorders (BPD) collection. To date, over 1,000 participants have been enrolled and genome-sequenced as part of the BRIDGE-BPD study for genome sequencing along with approximately 5,000 participants with other rare diseases enrolled to the NIHR BioResource–Rare Diseases (16). Two high-impact rare variants in *TPM4.1* and 1 in *TPM4.2* were identified. The variant affecting the product of *TPM4.2* was a nucleotide substitution of C to T at position 16192795 of chromosome 19 (build GRCh37). It was present on 1 allele in 2 unrelated BPD cases and was absent from the Exome Aggregation Consortium (ExAC) database (17) as well as the UK10K database (18). The 2 cases do not share any other rare variants, and their estimated genome-wide kinship, based on common unlinked SNVs (19), was estimated to be zero, suggesting that the variant did not arise in a recent common ancestor.

**Figure 2:**
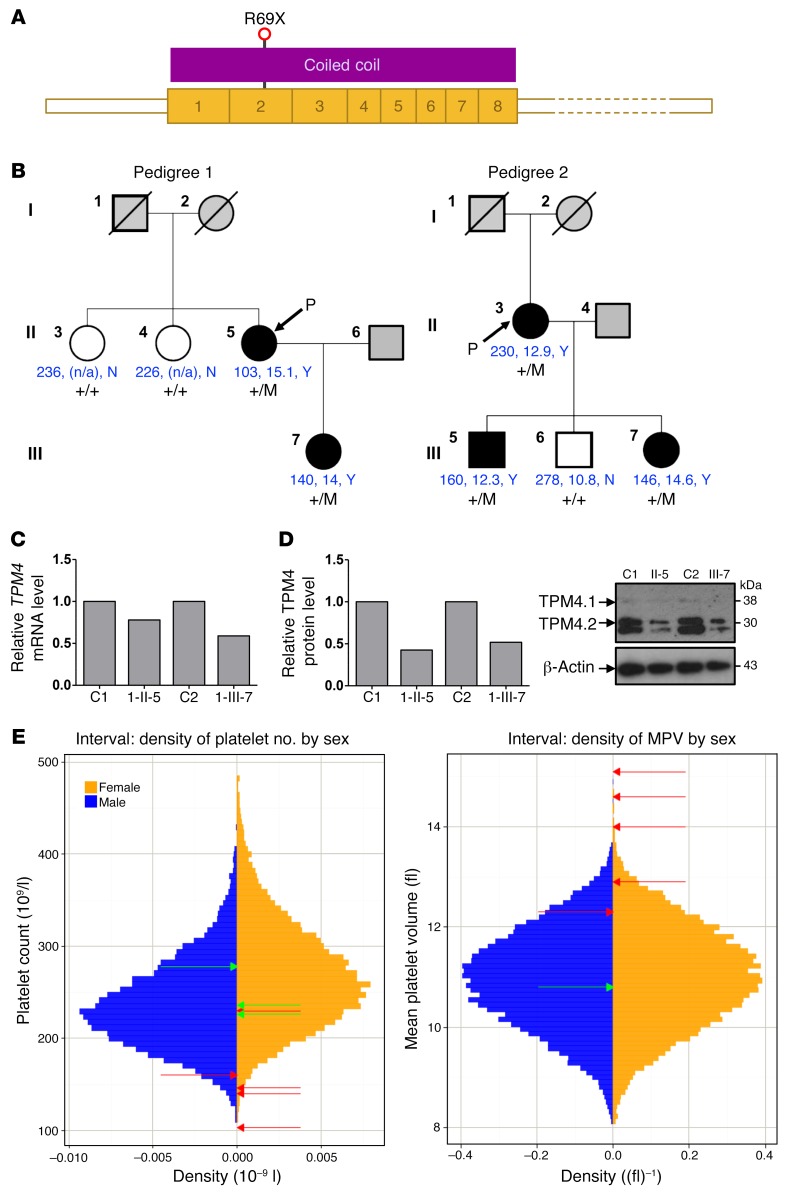
*TPM4* mutation causes macrothrombocytopenia in humans. (**A**) Schematic representation of the major megakaryocyte *TPM4* transcript ENST00000300933, which is predicted to encode the 248–amino acid TPM4 protein (UniProt ID P67936). R69 is transcribed from the second exon of the transcript and is 68 amino acids from the amino-terminus of TPM4. R69X is predicted to cause expression of a truncated TPM4 protein. (**B**) Family trees for pedigree 1 and pedigree 2 with the *TPM4* variant are depicted, including the platelet count, platelet volume, and presence/absence (Y/N) of macrothrombocytes when visualized by electron microscopy (shown in blue). Filled symbols: macrothrombocytopenia; gray symbols, unknown; open symbols: normal platelet count and volume and absence of macrothrombocytes. +/M, heterozygous; +/+, WT. (**C**) Platelet *TPM4* RNA levels measured by RT-PCR using *GAPDH* as housekeeping gene. Graph depicts representative data from a total of *n* = 3 measurements from 1 (case 1-II-5) or 3 (case 1-III-7) patient samples. (**D**) Platelet TPM4 protein is reduced in heterozygous carriers of the *TPM4* variant. Graph depicts representative data from a total of *n* = 3 measurements from 1 (case 1-II-5) or 3 (case 1-III-7) samples. Left: Densitometry analysis performed using ImageJ. Right: Protein levels of platelet TPM4 in controls and cases 1-II-5 and 1-III-7. β-Actin was included as an internal loading control. Similar results were obtained when GAPDH was used as a loading control (not shown). (**E**) Sex-stratified histograms of platelet count (PLT) and mean platelet volume measurements obtained using a Sysmex hematology analyzer from 48,345 blood donors from the INTERVAL study after adjustment for technical artifacts. The red arrows superimposed on the histograms indicate the sex of and values for patients with the truncating variant in *TPM4*. The green arrows indicate the sex of and values for relatives homozygous for the corresponding WT allele.

Predicted loss-of-function variants in TPM4.2 are rare in the general population, with only 2 heterozygotes observed among the more than 60,000 individuals in ExAC (1 with a splice site variant and 1 with a variant encoding a premature stop at residue 54). The variant concerning the BPD patients encodes a premature stop codon in exon 3 of *TPM4*, resulting in a truncation at residue 69 of the major protein isoform TPM4.2, and residue 105 of the minor isoform TPM4.1 (Figure 2A).

Macrothrombocytopenia was deemed present if the platelet count was below 150 × 10^9^ per liter or the mean platelet volume above 13 fl or if there was presence of macrothrombocytes as observed by electron microscopy. The propositus of pedigree 1 carrying the premature stop codon was a woman of mixed European descent who was enrolled to the BRIDGE-BPD study at 56 years old with a history of mild bleeding (see Supplemental Methods for full clinical history) consistent with observed platelet counts in the range of 80 × 10^9^ to 110 × 10^9^ per liter. Other than breast carcinoma, there was no other relevant past medical history. Analysis of 2 siblings and the propositus’ daughter demonstrated segregation of the observed variant with macrothrombocytopenia (Figure 2B and Supplemental Figure 1B). The propositus (1-II-5) and her daughter (1-III-7) exhibited platelet counts lower than those of the unaffected siblings (103 × 10^9^ and 140 × 10^9^ per liter compared with 226 × 10^9^ and 236 × 10^9^ per liter, respectively) with mean platelet volumes well above 13 fl. All other blood counts were within normal range (Table 1), suggesting a specific role for TPM4 in the megakaryocyte lineage.

The propositus of pedigree 2 was a woman of mixed European descent enrolled in the BRIDGE-BPD study at 58 years of age. She had a platelet count of 230 × 10^9^ per liter with a platelet volume near the upper limit of normal (12.9 fl). Macrothrombocytes were observed by electron microscopy. She had suffered with menorrhagia from menarche that did not improve upon hormonal therapy and therefore required recurrent dilatation and curettages, and finally a hysterectomy at the age of 37 (see Supplemental Methods for full clinical history). Platelet function tests including von Willebrand parameters and coagulation factors (IX, XI, and XIII) revealed no abnormality. Other than severe asthma and eczema, there was no past medical history of note. Analysis of 3 of the propositus’ children demonstrated segregation of the observed variant with a lower platelet count and larger platelets (Figure 2B and Supplemental Figure 1C). Pedigree member 2-III-5, who carries the variant, had a platelet count and mean platelet volume just within the normal range (160 × 10^9^ per liter and 12.3 fl, respectively). He experienced no abnormal bleeding in response to a number of hemostatic challenges, including a laparotomy, orchiopexy, and 4 dental extractions. His sibling (2-III-7), who also carries the mutation, exhibited a low platelet count (146 × 10^9^ per liter) and large mean platelet volume (14.6 fl), and a bleeding tendency similar to the propositus’ (see Supplemental Methods for full clinical history). 2-III-6 does not carry the variant and had the highest platelet count (278 × 10^9^ per liter) and lowest mean platelet volume (10.8 fl) of all family members. Thus, the variant cosegregates with isolated macrothrombocytopenia.

*TPM4.2* mRNA levels were reduced to 59% and 78% of the control in the affected cases 1-II-5 and 1-III-7, respectively (Figure 2C), and a corresponding approximately 50% reduction in the expression of TPM4.2 protein was observed with an antibody raised against the TPM4 C-terminus (δ/9d) (Figure 2D). Pedigree 2 exhibited similar defects, with mRNA levels reduced to 46%–63%, and we were able to detect a corresponding reduction in TPM4.2 protein in 2 of 3 affected pedigree members (Supplemental Figure 1D). The sex-stratified quantiles of mean platelet volume measurements with respect to the INTERVAL study (measurements obtained using a Sysmex hematology analyzer from 48,345 blood donors) were all above 91%, with 3 of the 5 measurements at percentiles exceeding 99.9% (20). This indicates that the platelets of the carriers are consistently large, with some extremely large. The sex-stratified quantiles of the platelet count (PLT) measurements with respect to INTERVAL were below 4% in all cases except the index case of pedigree 2, with 3 of the 5 measurements at percentiles below 1%. In contrast, all the WT homozygotes had PLT quantiles between 23% and 82% (Figure 2E). The probability of observing cosegregation under the null hypothesis that the mutant is not causal, conditional on having already observed the mutant in the propositi, is 0.5^3^ for each pedigree, and *P* = 0.0156 in total. Collectively, these data provide convincing evidence that loss-of-function variants in *TPM4* can cause macrothrombocytopenia, and suggest it plays a role in the development of the megakaryocyte lineage.

### A loss-of-function allele of murine Tpm4 causes macrothrombocytopenia.

**Figure 3:**
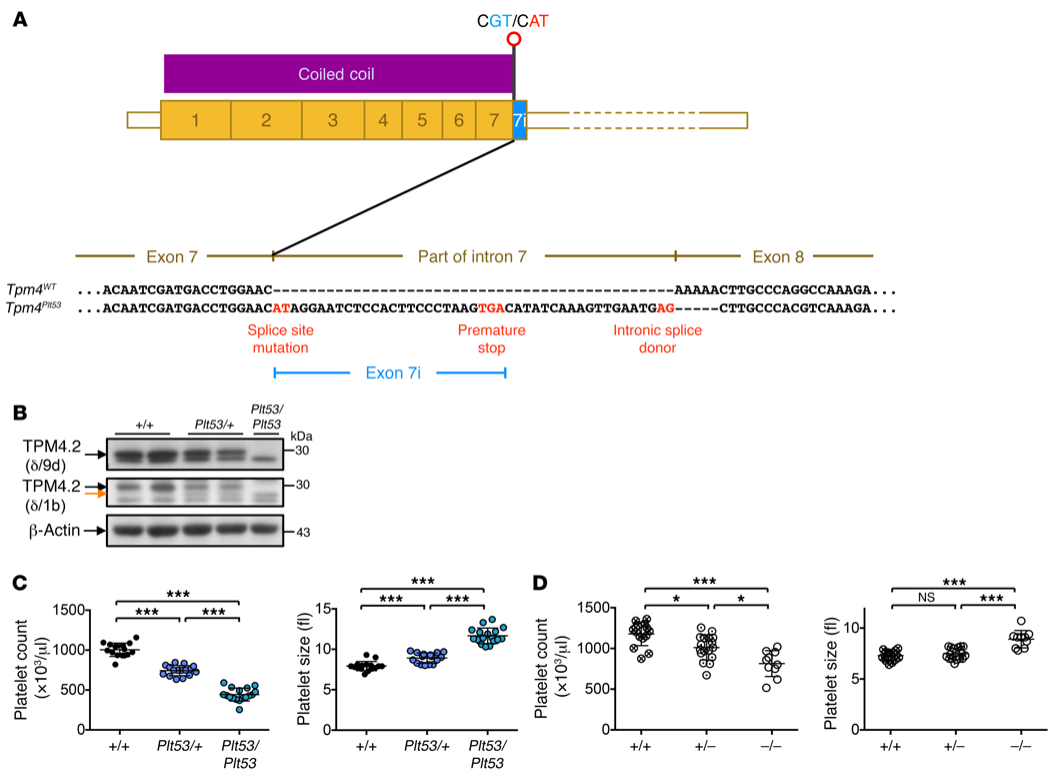
Loss of function at the *Tpm4* locus causes macrothrombocytopenia in mice. (**A**) Schematic representation of the *Plt53* mutation (A to G substitution at nucleotide g.72,147,268) in the first dinucleotide of the donor splice site in exon 7 of *Tpm4*, which is predicted to result in a protein bearing 8 intron-encoded amino acids followed by premature truncation due to the presence of an in-frame stop codon at nucleotide g.72,147,291. (**B**) Top panel: Use of an antibody directed against the TPM4 C-terminus (δ/9d, AB5449, Millipore) demonstrates that TPM4.2 is not expressed in *Tpm4^Plt53^* platelets. Middle panel: Use of an antibody directed against the TPM4 N-terminus (δ/1b) reveals residual expression of a truncated TPM4.2 protein from the *Tpm4^Plt53^* allele (orange arrow). Bottom panel: β-Actin loading control. Results are representative of 3 independent experiments. (**C**) The *Plt53* mutation causes macrothrombocytopenia. Reduced platelet count and increased platelet size in *Tpm4^Plt53/+^* and *Tpm4^Plt53/Plt53^* mice compared with *Tpm4^+/+^* mice (*n* = 17). (**D**) Platelet count and size in *Tpm4.2* WT (+/+), heterozygous (+/–), and homozygous knockout (–/–) mice (*n* = 10–15) on a C57BL/6 background. Measurements were performed using an Advia hematology analyzer. One-way ANOVA, unpaired 2-tailed Student’s *t* test with Bonferroni correction for multiple comparisons. *P < 0.05, ***P < 0.001.

In parallel studies, we conducted an *N*-ethyl-*N*-nitrosourea (ENU) mutagenesis screen in mice for mutations causing dominant thrombocytopenia (21, 22). A mouse exhibiting a decrease in platelet number and increase in mean platelet volume was identified and named *Plt53*. Fifty percent of the progeny from this animal also exhibited macrothrombocytopenia. After backcrossing, exome sequencing of 2 affected animals followed by genotyping of candidate mutations in siblings and offspring revealed that the *Plt53* phenotype correlated with inheritance of a point mutation in the gene encoding TPM4. The mutation, at nucleotide g.72,147,268, was a substitution of A for G in the first dinucleotide of the donor splice site in exon 7 of the *Tpm4* gene (hereafter referred to as *Tpm4^Plt53/Plt53^*). RT-PCR analysis of cDNA extracted from megakaryocytes revealed that the *Plt53* mutation led to the inclusion of part of intron 7 in the *Tpm4* mRNA. The predicted result is a protein bearing 8 intron-encoded amino acids followed by premature truncation due to the presence of an in-frame stop codon at nucleotide g.72,147,291 (Figure 3A).

Western blotting with an antibody raised against the TPM4 C-terminus (δ/9d) revealed the complete absence of TPM4 in *Tpm4^Plt53/Plt53^* platelets, and reduced expression in *Tpm4^Plt53/+^* platelets (Figure 3B, top). The lower band observed with this antibody was not detected with 2 other TPM4 antibodies and is therefore most likely unspecific (Supplemental Figure 2A). Using an N-terminal specific antibody (δ/1b), we observed a faint band of lower molecular weight in platelets from heterozygous and homozygous mutant mice, indicating the presence of some residual truncated TPM4 protein (Figure 3B, middle). Given that the overlap of N- and C-termini of TPM dimers is essential for filament formation (23), these data indicated that the *Plt53* allele of *Tpm4* represents a complete loss of the protein’s canonical function.

Heterozygous (*Tpm4^Plt53/+^*) and homozygous (*Tpm4^Plt53/Plt53^*) animals appeared healthy, and histopathological examination revealed no obvious organ abnormalities or pathology. Relative to WT littermates, *Tpm4^Plt53/+^* mice exhibited mild macrothrombocytopenia (Figure 3C), and *Tpm4^Plt53/Plt53^* animals a more severe form, with approximately 50% reduction in platelet counts. Remarkably, other peripheral blood cell counts in *Tpm4^Plt53/+^* and *Tpm4^Plt53/Plt53^* mice were unchanged, with the exception of a modest, but statistically significant (*P* = 0.0008), increase in lymphocyte counts in the *Tpm4^Plt53/Plt53^* cohort (Table 2). Flow cytometric analysis of bone marrow revealed normal numbers of immunophenotypic hematopoietic progenitor cells, including megakaryocyte progenitors, in *Tpm4^Plt53/Plt53^* mice (Table 3). Transplantation of unfractionated bone marrow from *Tpm4^Plt53/Plt53^* animals into lethally irradiated adult recipients resulted in macrothrombocytopenia (Supplemental Figure 2B), demonstrating that the phenotype was intrinsic to the hematopoietic system. To confirm the *Plt53* mutation as the causative genetic lesion, we generated an independent knockout mouse strain for TPM4.2, the only TPM4 protein isoform expressed in mice. Strikingly, given the early lethality observed in *Tpm1*, *Tpm2*, and *Tpm3* knockouts, animals lacking *Tpm4* were viable and outwardly healthy. They exhibited a dose-dependent macrothrombocytopenia (Figure 3D and Supplemental Figure 3, A–C), similar in magnitude to that observed in *Tpm4^Plt53^* mutants on a C57BL/6 background (the background on which the targeted knockout allele was generated) (Supplemental Figure 3D). Together, these data demonstrate a specific requirement for TPM4 in the megakaryocyte lineage.

### TPM4 insufficiency results in aberrant platelet morphology.

**Figure 4:**
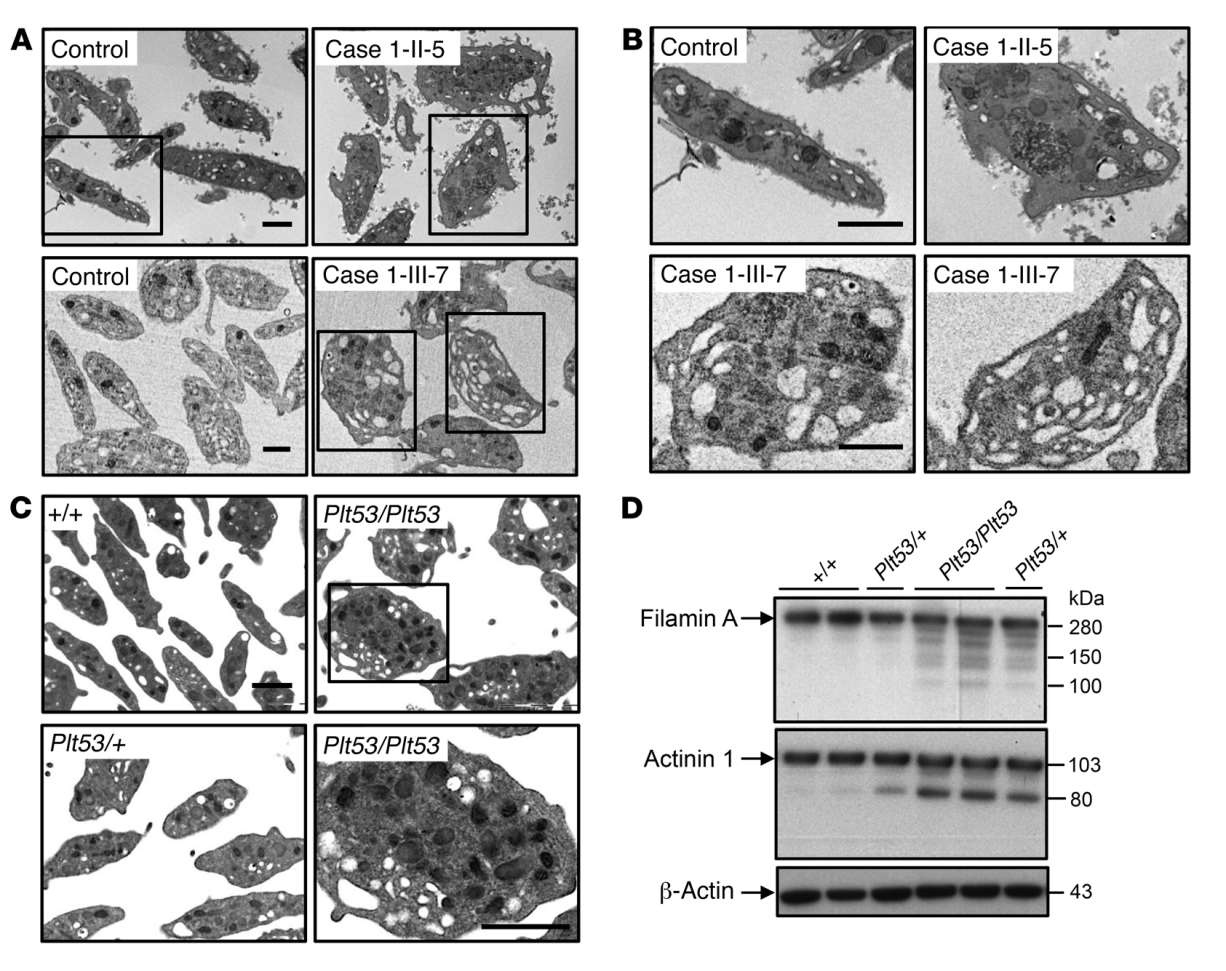
TPM4 insufficiency results in altered platelet morphology. (**A** and **B**) Ultrastructure of platelets from cases 1-II-5 and 1-III-7 carrying the variant, showing the presence of large platelets with numerous vacuoles indicating increased fragility, contrasting the normal discoid platelet appearance in controls. (**A**) Overview. (**B**) Detail. Scale bars: 1 μm. (**C**) Representative electron microscopic pictures illustrating increased size and fragile appearance in *Tpm4^Plt53/Plt53^* and *Tpm4^Plt53/+^* compared with *Tpm4^+/+^* platelets (*n* = 2; each sample was pooled from 2 individuals). Scale bars: 1 μm. (**D**) Western blot showing the presence of degraded filamin A and actinin 1 in *Tpm4^Plt53/Plt53^* and *Tpm4^Plt53/+^* compared with *Tpm4^+/+^* platelets. Results are representative of 2 independent experiments. Bottom panel: β-Actin loading control.

Transmission electron microscopy (TEM) analysis of platelets from the 2 affected cases from pedigree 1 revealed considerable heterogeneity in platelet size, some of them being very large. We also observed an increased presence of large vacuoles (Figure 4, A and B). Blinded analysis of TEM pictures correctly identified all the carriers from pedigree 2. Platelets from 2-II-3 and 2-III-5 exhibited moderate defects (Supplemental Figure 4) similar to those observed for pedigree 1 (Figure 4, A and B). Platelets from 2-III-7 were obviously different from the control, with an increased small diameter and an ellipsoid rather than discoid shape.

Consistent with the human data, TEM analysis revealed considerable heterogeneity in the size and morphology of *Tpm4^Plt53/Plt53^* and *Tpm4^Plt53/+^* platelets, with frequent vacuoles, indicating increased fragility of the mutant platelets (Figure 4C). Given that ultra-rare variants in *FLNA* (the gene encoding filamin A) have recently been reported to result in fragile platelets and macrothrombocytopenia (24), and variants in the actin–cross-linking protein actinin 1 (α-actinin, *ACTN1*) are one of the most frequent causes of isolated thrombocytopenia with altered platelet morphology (25–27), we examined the levels of these 2 proteins. Interestingly, we found that filamin A was partially degraded in *Tpm4^Plt53/Plt53^* and to a lesser extent *Tpm4^Plt53/+^* platelets (Figure 4D, top). Levels of full-length actinin 1 were reduced in *Tpm4^Plt53/Plt53^* and *Tpm4^Plt53/+^* platelets, coinciding with the appearance of an 80-kDa band that has been previously reported to be the result of cleavage by calpain (Figure 4D, bottom) (28).

### Reduced TPM4 expression impacts platelet function.

**Figure 5:**
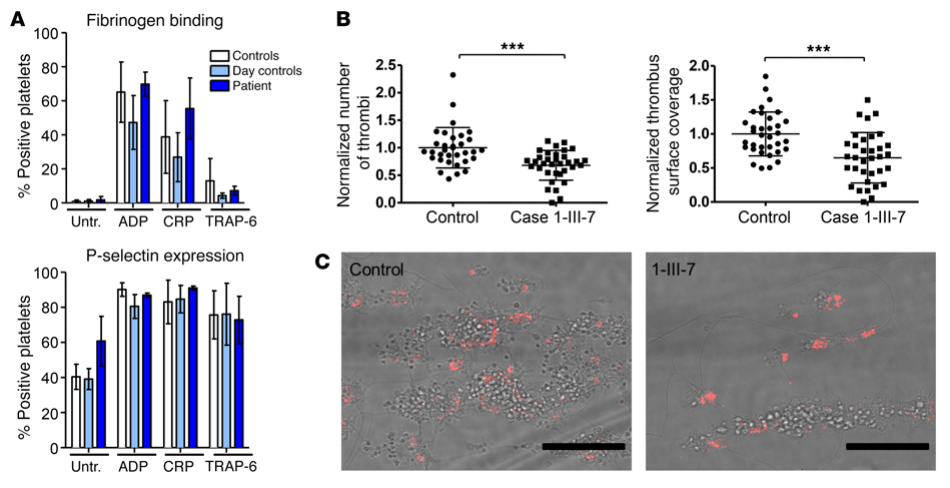
The function of human platelets is mildly affected by reduced TPM4 expression. (**A**) Platelet function testing measuring fibrinogen binding (top) and P-selectin expression (bottom) (percentage of positive platelets) after treatment with final concentrations of 0.0005 μM ADP, 0.3 μg/ml CRP-XL, or 0.8 μM TRAP-6. Depicted are the results obtained on 3 different days (2 technical replicates) for the day control and the patient (case 1-III-7). A bank of 20 controls is shown in white. (**B** and **C**) Thrombus formation on collagen under flow (shear rate 1,600/s). (**B**) Normalized thrombus number and coverage after blood perfusion through a collagen-coated chamber; 6 images captured per run, 2 runs per sample using 2 different samples. (**C**) Representative images of thrombi stained with P-selectin captured using fluorescence microscopy (EVOS system; Advanced Microscopy Group). Scale bars: 50 μm. Unpaired 2-tailed Student’s *t* test, ***P < 0.001.

We next examined the function of the platelets of the daughter of the propositus of pedigree 1 (case 1-III-7 in Figure 2B). In response to any of the agonists ADP, cross-linked collagen-related peptide (CRP-XL), and TRAP-6, no significant differences in platelet activation measured by integrin α_IIb_β_3_ activation and α-granule secretion were revealed (Figure 5A). However, when thrombus formation under flow ex vivo was examined, both the number of thrombi and the area covered were reduced in case 1-III-7 relative to the control (*P* < 0.001) (Figure 5, B and C).

**Figure 6:**
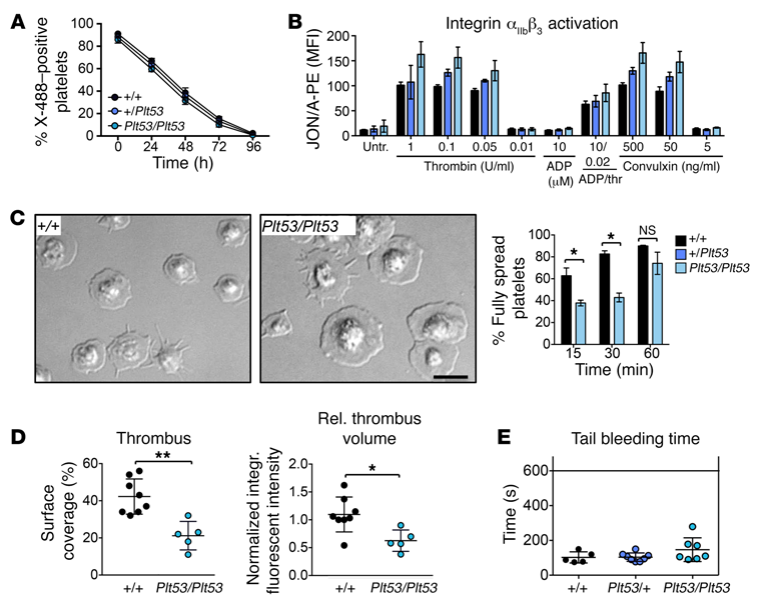
The function of mouse platelets is mildly affected by reduced TPM4 expression. (**A**) Normal in vivo lifespan of *Tpm4^Plt53/+^* and *Tpm4^Plt53/Plt53^* compared with *Tpm4^+/+^* platelets. Platelets were labeled by i.v. injection with X-488 antibody (Emfret), and the percentage of fluorescent platelets was monitored over time by flow cytometry (*n* = 5, representative of 2 independent experiments). (**B**) Flow cytometric measurement of integrin α_IIb_β_3_ activation (JON/A–PE antibody) in *Tpm4^+/+^*, *Tpm4^Plt53/+^*, and *Tpm4^Plt53/Plt53^* platelets after activation with the depicted agonists (*n* = 4, representative of 3 independent experiments). (**C**) Platelet spreading. Left: Representative differential interference microscopy images of *Tpm4^+/+^* and *Tpm4^Plt53/Plt53^* platelets spread on fibrinogen (100 μg/ml) after activation with 0.01 U/ml thrombin. Scale bar: 5 μm. Right: Percentage of fully spread *Tpm4^+/+^* (black) and *Tpm4^Plt53/Plt53^* (blue) platelets at 15, 30, and 60 minutes after induction of the spreading process (*n* = 3). Results are representative of 3 independent experiments. (**D**) Thrombus formation on collagen under flow (shear rate 1,000/s). Left: Mean surface covered with thrombi. Right: Relative platelet deposition, as measured by integrated fluorescent intensity per square millimeter ± SD. Each dot represents an individual. Results are pooled from 2 independent experiments. (**E**) Tail bleeding times in *Tpm4^Plt53/+^* (blue) and *Tpm4^Plt53/Plt53^* (light blue) compared with *Tpm4^+/+^* (black) mice. Each dot represents an individual. Unpaired 2-tailed Student’s *t* test, *P < 0.05, **P < 0.01.

We then probed the impact of TPM4 dysfunction on platelet function in mice. Given that macrothrombocytopenia can be triggered by accelerated platelet clearance, we firstly examined the in vivo lifespan of *Tpm4^+/+^*, *Tpm4^Plt53/+^*, and *Tpm4^Plt53/Plt53^* platelets. No change in the kinetics of clearance or ultimate lifespan was observed, indicating that lack of functional TPM4 did not affect platelet turnover (Figure 6A). Platelet activation was assessed in vitro by flow cytometry using integrin α_IIb_β_3_ activation (JON/A antibody) and α-granule secretion (anti–P-selectin antibody) as markers. *Tpm4^Plt53/+^* and *Tpm4^Plt53/Plt53^* platelets were responsive to a range of agonists, indicating that the processes of integrin inside-out activation and degranulation are functional in the absence of TPM4 (Figure 6B and Supplemental Figure 5A). The increased mean fluorescence intensity for integrin α_IIb_β_3_ in the *Tpm4^Plt53/Plt53^* samples was most probably due to the increased size of the mutant platelets, as it correlated with an approximately 37% increase in basal CD41 (integrin α_IIb_ subunit) fluorescence signal (Figure 6B and Supplemental Table 1). To investigate the involvement of TPM4 in activation-induced morphological changes, we incubated thrombin-activated *Tpm4^+/+^* and *Tpm4^Plt53/Plt53^* platelets on fibrinogen-coated coverslips. Under these conditions, platelets from both genotypes fully spread, but the spreading process was significantly delayed in the mutant (Figure 6C). As seen with case 1-III-7, thrombus formation on collagen under flow ex vivo was significantly reduced in blood from *Tpm4^Plt53/Plt53^* mice (Figure 6D and Supplemental Figure 5B). Finally, we found that tail bleeding times were mildly extended in *Tpm4^Plt53/Plt53^* mice, and in lethally irradiated C57BL/6 mice with transplanted *Plt53/Plt53* bone marrow, relative to their respective controls (*P* = 0.165 and *P* = 0.045; Figure 6E and Supplemental Figure 5C). Together, these results demonstrate that the hemostatic function of TPM4-deficient platelets is mildly compromised.

### TPM4 localizes to proplatelets in human and mouse megakaryocytes.

**Figure 7:**
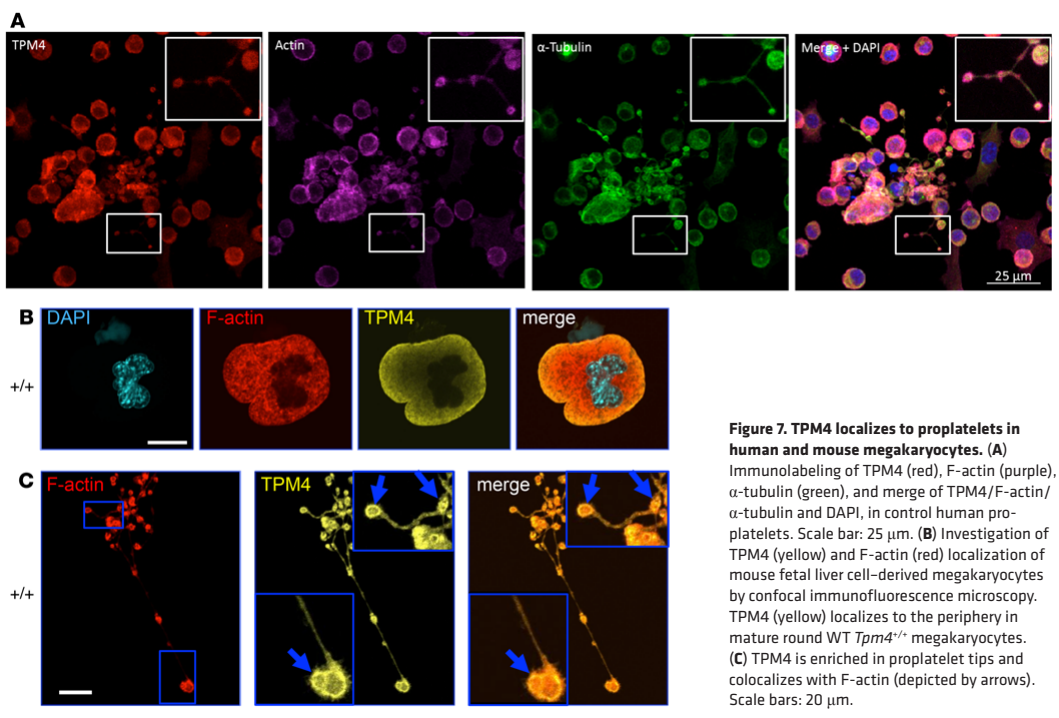
TPM4 localizes to proplatelets in human and mouse megakaryocytes. (**A**) Immunolabeling of TPM4 (red), F-actin (purple), α-tubulin (green), and merge of TPM4/F-actin/α-tubulin and DAPI, in control human proplatelets. Scale bar: 25 μm. (**B**) Investigation of TPM4 (yellow) and F-actin (red) localization of mouse fetal liver cell–derived megakaryocytes by confocal immunofluorescence microscopy. TPM4 (yellow) localizes to the periphery in mature round WT *Tpm4^+/+^* megakaryocytes. (**C**) TPM4 is enriched in proplatelet tips and colocalizes with F-actin (depicted by arrows). Scale bars: 20 μm.

Sorting of tropomyosin isoforms to different cellular compartments is thought to be crucial for their function (9). TPM4 localized to proplatelets and colocalized with filamentous (F) actin in primary CD34^+^-derived human megakaryocytes (Figure 7A). In mature, WT mouse megakaryocytes, TPM4 showed a diffuse, mainly peripheral distribution and did not obviously colocalize with F-actin (Figure 7B). As expected, we could not detect TPM4 in *Tpm4^Plt53/Plt53^* megakaryocytes using the δ/9d antibody (Supplemental Figure 6A). The distribution and morphology of F-actin and tubulin were similar in mature WT and *Tpm4^Plt53/Plt53^* megakaryocytes (not shown). In WT megakaryocytes commencing proplatelet formation, TPM4 was organized in a fine punctuate network (Supplemental Figure 6B). TPM4 colocalized with actin in proplatelet swellings and tips and partially at proplatelet membranes (Figure 7C).

### TPM4 facilitates proplatelet formation in human and mouse.

**Figure 8:**
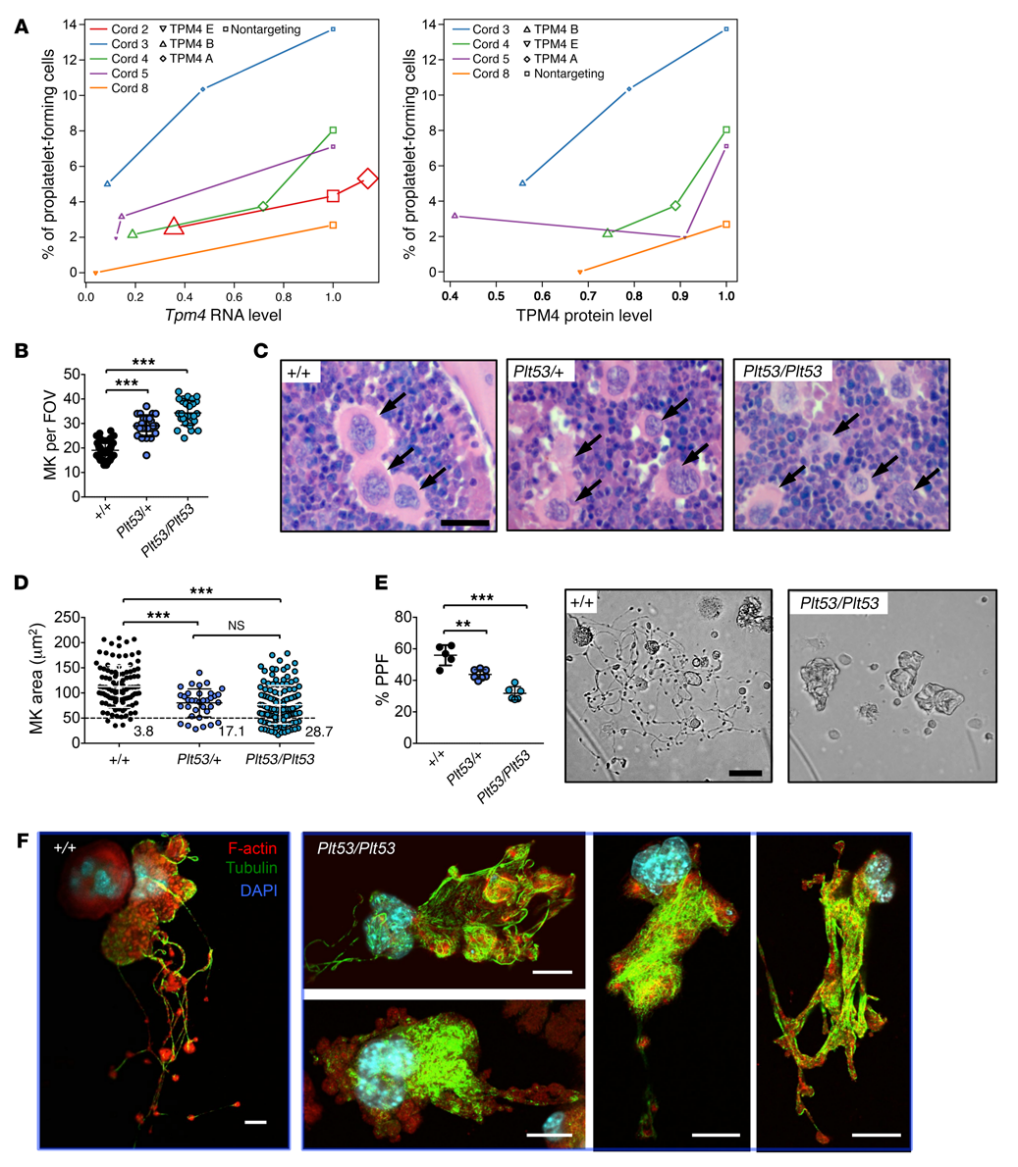
TPM4 dose-dependently facilitates proplatelet formation. (**A**) Logistic regression analysis showing that the percentage of proplatelet-forming cells decreases with *Tpm4* RNA (left; effect 1.22, *P* < 2^–16^) and protein (right; effect 2.54, *P* = 6.23^–12^) levels. Node size reflects the number of cells counted (at least 200). (**B**) Increased number of megakaryocytes (MK) in bone marrow from *Tpm4^Plt53/+^* and *Tpm4^Plt53/Plt53^* mice. Shown are results from 50 fields of view (FOV) (*Tpm4^+/+^* and *Tpm4^Plt53/+^*) and 28 FOV (*Tpm4^Plt53/Plt53^*). (**C**) Representative pictures of H&E-stained bone marrow sections show altered morphology (smaller size, irregular shape) of *Tpm4^Plt53/+^* and *Tpm4^Plt53/Plt53^* megakaryocytes compared with the control (*n* = 3). Arrows indicate megakaryocytes. Scale bar: 15 μm. (**D**) Decreased size of *Tpm4* mutant megakaryocytes compared with WT counterparts. *n* = 105 (*Tpm4^+/+^*), *n* = 35 (*Tpm4^Plt53/+^*), *n* = 125 (*Tpm4^Plt53/Plt53^*). (**E**) Left: Decreased proplatelet formation (PPF) of fetal liver cell–derived *Tpm4^Plt53/+^* and *Tpm4^Plt53/Plt53^* megakaryocytes (*n* = 5–6). Each dot represents the mean of 1 individual sample (at least 12 visual fields counted). Right: Representative light microscopy pictures showing altered morphology and decreased branch formation of *Tpm4^Plt53/+^* and *Tpm4^Plt53/Plt53^* compared with *Tpm4^+/+^* megakaryocytes. Scale bar: 50 μm. (**F**) Investigation of F-actin (red) and tubulin (green) distribution of proplatelet-forming *Tpm4^+/+^* (left) and *Tpm4^Plt53/Plt53^* (right) megakaryocytes by confocal immunofluorescence microscopy. Nuclei were stained with DAPI (blue). Scale bars: 20 μm. (**B** and **E**) One-way ANOVA, unpaired 2-tailed Student’s *t* test with Bonferroni correction for multiple comparisons. (**D**) One-way ANOVA, Mann-Whitney test with Bonferroni correction for multiple comparisons. **P < 0.01, ***P < 0.001.

We next examined the effect of depleting *TPM4* in human megakaryocytes derived from CD34^+^ hematopoietic progenitor cells. While megakaryocyte proliferation and maturation were unaffected by transduction with shRNAs targeting *TPM4* (Supplemental Figure 7A), the level of proplatelet formation was reduced in comparison with the nontargeting control. ShRNA_*TPM4B* and shRNA_*TPM4E*, which knocked down mRNA levels to approximately 20% and 8%, respectively, reduced proplatelet formation by approximately 40% and 14%. Logistic regression analysis showed that the correlation between the proportion of proplatelet-forming cells and RNA and protein levels for TPM4 was highly significant (*P* < 2^–16^ and < 6.23^–12^, respectively) (Figure 8A), demonstrating that the level of TPM4 is rate-limiting for platelet formation.

In mice, we observed a significant increase in megakaryocyte numbers in *Tpm4^Plt53/+^* and *Tpm4^Plt53/Plt53^* bone marrow (Figure 8B). Ploidy profiles were unchanged (Supplemental Figure 7B). However, mutant megakaryocytes were smaller than WT counterparts (~20% of *Tpm4^Plt53/+^* and ~30% of *Tpm4^Plt53/Plt53^* compared with only ~4% of *Tpm4^+/+^* megakaryocytes were smaller than 50 μm^2^), and many exhibited an irregular morphology (Figure 8, C and D). Relative to the WT, proplatelet formation was reduced by approximately 40% in fetal liver–derived *Tpm4^Plt53/Plt53^* megakaryocytes and by approximately 20% in *Tpm4^Plt53/+^* cells (Figure 8E). Time-lapse video microscopy indicated that, while the initial morphological changes associated with proplatelet formation took place in mutant cells, loss of TPM4 resulted in megakaryocytes with few or no branches (Figure 8F and Supplemental Videos 1 and 2). Where proplatelets did form, their tips were significantly increased in size relative to WT counterparts (Supplemental Figure 7C). Visualization of F-actin and tubulin demonstrated that within the short, thick branches produced by *Tpm4^+/+^* megakaryocytes, massive networks of tubulin were diffusely distributed throughout the cell (Figure 8F). Collectively, these data demonstrate an essential role for TPM4 in platelet shedding and sizing.

### Potential TPM4-interacting proteins in megakaryocytes and platelets.

**Figure 9:**
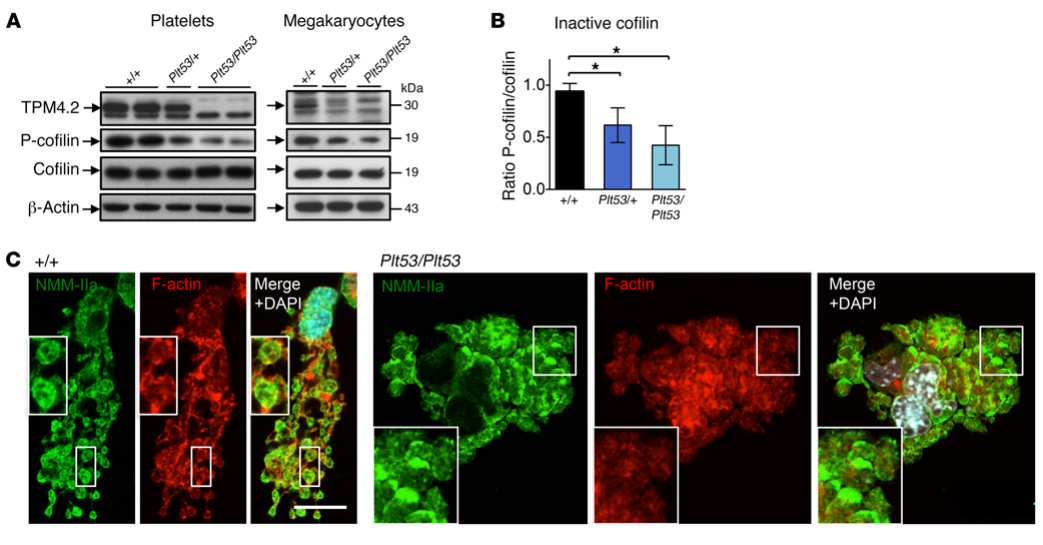
TPM4-interacting proteins in megakaryocytes and platelets. (**A**) Investigation of cofilin and P-cofilin expression in *Tpm4^Plt53/Plt53^* platelets (left) and fetal liver cell–derived megakaryocytes (right) by Western blot. Blots are representative of 2–3 individual experiments. (**B**) Densitometry analysis shows decreased levels of phosphorylated (inactive) cofilin in *Tpm4^Plt53/+^* and *Tpm4^Plt53/Plt53^* compared with *Tpm4^+/+^* platelets (*n* = 6; results were pooled from 2 separate experiments). (**C**) Investigation of NMMHC-IIa (green) and F-actin (red) localization in fetal liver cell–derived *Tpm4^+/+^* and *Tpm4^Plt53/Plt53^* megakaryocytes by confocal immunofluorescence microscopy. Scale bar: 20 μm. One-way ANOVA, unpaired 2-tailed Student’s *t* test with Bonferroni correction for multiple comparisons. *P < 0.05.

Tropomyosins have been reported to interact with several proteins that regulate platelet biogenesis. We examined the actin filament–severing proteins actin-depolymerizing factor (ADF) and cofilin, as these have been shown to directly compete with tropomyosins for actin binding (29). While ADF expression was unaltered in *Tpm4* mutant mouse megakaryocytes and platelets (data not shown), the levels of phosphorylated (inactive) cofilin in *Tpm4^Plt53/Plt53^* cells were significantly decreased (Figure 9, A and B). This suggested enhanced actin turnover in mutant cells. Consistent with this, a reduction in F-actin levels was observed in nonstimulated mutant platelets (Supplemental Figure 8).

Given that TPM4 localization was reported to be abnormal in proplatelet tips deficient in tropomodulin 3 (TMOD3) (30), we investigated TMOD3 localization in cultured *Tpm4^+/+^* and *Tpm4^Plt53/Plt53^* megakaryocytes. In line with that study, we observed that TMOD3 localized as puncta with proplatelet membranes (Supplemental Figure 9). While TMOD3 appeared to be more diffusely distributed in some *Tpm4^Plt53/Plt53^* proplatelets, we did not detect major alterations between the mutant and control samples. Besides their well-known interaction with tropomodulins, tropomyosins have also been demonstrated to recruit myosin IIa to stress fibers, and to be involved in stabilization of RHOA (31, 32). Inherited mutations in the *MYH9* gene, which encodes nonmuscle myosin heavy chain IIa (NMMHC-IIa), give rise to the “MYH9-related disorders,” all of which exhibit macrothrombocytopenia, and have been linked to the RHOA/ROCK pathway (33). We therefore examined the expression and phosphorylation state of RHOA, ROCK, and NMMHC-IIa by Western blot. No differences between *Tpm4^Plt53/Plt53^*, *Tpm4^Plt53/+^*, and *Tpm4^+/+^* megakaryocytes and platelets were observed (Supplemental Figure 10A). Confocal microscopy of *Tpm4^+/+^* megakaryocytes undergoing the first morphological changes presaging proplatelet formation revealed that NMMHC-IIa localized to a fine network (Supplemental Figure 10B), very similar to the distribution of TPM4 (Supplemental Figure 6B). This pattern was dramatically perturbed in approximately 50% of *Tpm4^Plt53/Plt53^* megakaryocytes. In WT proplatelet swellings and tips, NMMHC-IIa localized to a previously described characteristic network and to membranes (Figure 9C, left, and ref. 34). In contrast, NMMHC-IIa in *Tpm4^Plt53/Plt53^* proplatelet-forming megakaryocytes showed a patchy distribution in approximately 70% of cells analyzed, as compared with approximately 15% of *Tpm4^+/+^* counterparts (Figure 9C, right). These results indicate that TPM4 deficiency impacts multiple regulators of the megakaryocyte actin cytoskeleton.

## Discussion

Cytoskeletal reorganizations are the driving force for the production of platelets by megakaryocytes (35). Here, we show that *TPM4*, previously identified by genome-wide association studies as a candidate gene correlated to platelet count and volume, is a modulator of platelet production. *TPM4* insufficiency causes macrothrombocytopenia in mice and humans in a dose-dependent manner.

The tropomyosin genes and particularly *TPM4* are highly expressed in human and murine megakaryocytes, and the corresponding proteins are abundant in platelets (Figure 1). Our study demonstrates that relatively modest alterations in *TPM4* dosage are sufficient to impact platelet biogenesis. The level of *TPM4* expression in megakaryocytes determines their ability to produce correctly sized platelets. Our findings strongly suggest that TPM4 localization and interaction with actin filaments and other cytoskeletal components facilitate megakaryocyte branching during proplatelet formation. It has been previously reported that TPM4.2 overexpression in a neuronal cell line augmented branch formation (36). Both proplatelet formation and neurite branching require extensive localized cytoskeleton-membrane interactions. In line with this, recent reports have shown that TPM4.2-bound actin filaments are found at membrane regions with specialized functions, such as the postsynaptic membrane (37), the external face of the sarcoplasmic reticulum (38), or podosomes (39). Our findings therefore suggest that the amount of available TPM4-bound actin filaments in megakaryocytes directly determines the amount of membrane regions, which can generate proplatelet branches.

Our results indicate that TPM4 insufficiency affects multiple cytoskeletal regulators with known roles in platelet production, including cofilin, myosin IIa, actinin 1, and filamin A. The abnormal distribution of tubulin, polymerization of which is considered a driving force for proplatelet elongation (2, 40), was potentially a downstream effect. Despite recent work suggesting that capping of TPM4-bound actin filaments by TMOD3 is required for proplatelet formation (30), TMOD3 localization was not obviously affected in *Tpm4* mutant proplatelets. This might be explained by TPM1 or TPM3.1 partially compensating for the lack of TPM4 during proplatelet formation.

Mice lacking cofilin in the megakaryocyte lineage display macrothrombocytopenia (41), suggesting that the correct balance between TPM4 and cofilin is essential for the production of correctly sized platelets. The decreased levels of inactive, phosphorylated, cofilin in *Tpm4* mutant megakaryocytes and platelets are consistent with previous studies demonstrating that tropomyosin and cofilin compete with each other at the actin filament (29), and indicate that reduced TPM4 expression facilitates increased binding of active cofilin to actin filaments. Mutations in the gene encoding NMMHC-IIa (*MYH9*) in cases with MYH9-related disorders cause autosomal dominant macrothrombocytopenia and bleeding associated with other complex pathobiologies (42). Altered localization of NMMHC-IIa was observed in a large proportion of *Tpm4* mutant megakaryocytes. This finding is in accordance with a previously established role for TPM4.2 in myosin II recruitment to stress fiber precursors (31) and its specific regulatory impact on NMMHC-IIa (43).

Reduced TPM4 expression resulted in the production of large platelets with fragile appearance (Figure 4 and Supplemental Figure 4). Fragile platelets with impaired function are a characteristic of patients with *FLNA* mutations (24, 44), and of *Flna*-null mice (45). We observed increased levels of degraded filamin A in *Tpm4* mutant platelets. Interaction of filamin A with glycoprotein (GP1B), a component of the VWF receptor complex, is important for regulation of platelet size, as well as platelet adhesion and maintenance of membrane integrity under high shear (46–48). Thus, disturbances in interactions between filamin A and F-actin may contribute to the altered size and function of TPM4-deficient platelets. *Tpm4* mutant platelets also exhibited partial degradation of actinin 1. Mutations in the latter cause autosomal dominant thrombocytopenia (25–27). Prior studies have suggested that the levels of actin-binding proteins correlate with fluctuations in tropomyosin isoforms (36, 49). Our work indicates that loss of TPM4 binding inhibits binding of other proteins, such as filamin A and actinin 1, to actin filaments. This results in an impairment of the complex actomyosin cytoskeletal network that drives proplatelet formation.

TPM4 insufficiency affected neither the in vivo platelet lifespan in mice, nor the in vitro platelet functional responses to agonists in humans and mice. The delayed spreading observed in *Tpm4* mutant platelets may be attributed to their increased size, but may also indicate specific defects upon decreased TPM4 expression, potentially involving altered integrin α_IIb_β_3_ outside-in signaling (Figure 7C). Consistent with this, mutant mice, and WT mice with transplanted mutant bone marrow, tended toward prolonged tail bleeding times. Furthermore, thrombus formation on collagen under flow by TPM4-deficient platelets of both human and mouse origin was reduced. Since the platelet disorder associated with the *TPM4* variant results in an unpredictable bleeding phenotype, these findings indicate that reduced levels of TPM4 result in a mild impairment of hemostasis.

Our findings indicate that, like the targeted knockout, the *TPM4* variants we identified in both humans and mice result in a complete loss of function at the *TPM4* locus. The fact that mice homozygous for the *Plt53* mutation or null allele are healthy and fertile was very surprising, standing in stark contrast to mice lacking *Tpm1*, *Tpm2*, or *Tpm3*, which die in utero (11–13). Thus, TPM4 is the only tropomyosin family member dispensable for development. It plays an essential and highly specific role in F-actin organization in the megakaryocyte lineage. Given the exceptionally high level of conservation between them, the inability of other tropomyosin isoforms to fully compensate for the loss of *TPM4* deletion highlights a profound gap in our understanding of how functional specificity is achieved and governed.

## Methods

### Mice

#### Generation of mice with a mutant Tpm4 allele.

Mice carrying a mutant *Tpm4* allele, named *Plt53*, were generated. Briefly, male BALB/c mice were injected i.p. with 85 mg/kg ENU weekly for 3 weeks (50). Treated animals were rested for 12 weeks before mating with untreated BALB/c females to produce first-generation progeny. Peripheral blood was taken from these mice at 7 weeks of age and a complete blood cell count performed using an Advia 2120 hematological analyzer. Animals exhibiting aberrant platelet counts were test-mated to determine heritability of the phenotype. Mutant strains were backcrossed for 10 generations to the BALB/c background in order to breed out irrelevant ENU-induced mutations. *Tpm4^Plt53/+^* mice were intercrossed to yield *Tpm4^+/+^*, *Tpm4^Plt53/+^*, and *Tpm4^Plt53/Plt53^* littermates, which were used in experiments. Mice were 7 to 12 weeks old or as otherwise stated.

#### Generation of Tpm4.2 knockout mice.

Target sites in exon 1b of the mouse *Tpm4* gene were analyzed by the MIT CRISPR Design Tool (51). Two high-scoring (>85) guide RNA (gRNA) sequences were chosen, as they created a 93-bp deletion that interrupted the ATG start codon. The mouse oocytes were preincubated in cytochalasin B (5 μg/ml); then 12.5 ng/μl of each commercially synthesized gRNA (Sigma-Aldrich) and 50 ng/μl of Cas9 mRNA were coinjected into the cytoplasm of mouse oocytes (52). Progeny were screened for the 93-bp deletion using the primers *Tpm4.2* total KO forward 5′-GTGACCTCATGGGCCTGAC-3′ and *Tpm4.2* total KO reverse 5′-GGACGAAAAGTGGGATCG-3′, which detects a WT allele of 317 bp and a knockout allele of 223 bp. DNA from founder mouse 6 had the expected 90-bp deletion, and a subsequent Western blot of tail lysates revealed the complete absence of TPM4.2, the sole TPM4 protein isoform expressed in mice.

### Hematology

#### Human.

Automated full blood cell counts were performed on blood collected by venipuncture into tubes containing EDTA using a Sysmex XN-1000 or XE-5000 hematological analyzer.

#### Mouse.

Automated cell counts were performed on blood collected by cardiac puncture or from the retro-orbital plexus into Microtainer tubes containing EDTA (Sarstedt), using an Advia 2120 hematological analyzer (Siemens). Megakaryocytes were counted manually in sections of sternum stained with H&E with a minimum of 10 visual fields (×200) analyzed. Images were acquired on a Nikon Eclipse E600 microscope equipped with AxioCam MRc5 (Zeiss) and AxioVision 4.8. Scale bars were inserted and the megakaryocyte area was determined using ImageJ (NIH). Acute thrombocytopenia was induced by injection of antiplatelet serum and assessed as previously described (53).

### Enumeration of hematopoietic progenitors in mice by flow cytometry

Organ cellularity was determined by counting of cell suspensions from bone marrow, spleen, and thymus with calibrate beads (BD Biosciences). Cells were stained on ice for 30 minutes, washed once, and then incubated with streptavidin–PE–Texas red (Life Technologies). After a washing step, acquisition was performed on an LSR Fortessa 1 (BD Biosciences) and analyzed with FlowJo (Tree Star). Antibodies for flow cytometry either were labeled in house with FITC, PE, PECy7, APC, or Alexa Fluor 700 or were biotinylated. CD4 (GK1.5), CD8 (53.6.7), CD16/32 (24G2), CD19 (1D3), CD41 (MWReg30), Ter119 (Ly76), Gr1 (RB6-8C5), CD11b (M1/70), Sca1 (D7), Kit (ACK4), B220 (RA3-6B2), and CD34-FITC (RAM34) were purchased from BD Pharmingen, and CD150-PE (TC15-12F12.2) was purchased from BioLegend. Fluorogold (Sigma-Aldrich) was used to exclude dead cells.

### Investigation of the effect of the *Plt53* mutation in megakaryocytes by RT-PCR

Fetal liver cell–derived megakaryocytes from *Tpm4^+/+^*, *Tpm4^Plt53/+^*, and *Tpm4^Plt53/Plt53^* embryos E13.5 were generated as described below. mRNA was isolated using the RNeasy Plus Kit (Qiagen), and cDNA was synthesized using SuperScript III Reverse Transcriptase (Invitrogen) according to the manufacturer’s protocols. The primers used were 5′-AAAGAAGCCAAGCACATCAC-3′ (Tm4_F3) and 5′-AGCCCACATTCTCTTCTTTG-3′ (Tm4_R7). The expected product size for the WT was 360 bp, while an approximately 400-bp product was obtained in the presence of the *Tpm4^Plt53^* allele. PCR products were sequenced and revealed the presence of an approximately 40-bp intronic inclusion in the mutant compared with the WT product.

### Gene expression analysis in human megakaryocytes by RT–quantitative PCR

Total RNA was extracted using an RNeasy Plus Kit (Qiagen) according to the manufacturer’s instructions. cDNA was prepared from 250–500 ng total RNA using SuperScript III First-Strand Synthesis system for RT-PCR (Invitrogen) with random hexamers. Two-step quantitative PCR (qPCR) reactions were performed in duplicates using SYBR Green chemistry (Brilliant II SYBR Green QPCR Low ROX, Agilent Technologies) on an Mx3000P instrument (Agilent Technologies). Relative gene expression was calculated by the ΔΔCt method using *GAPDH* housekeeping gene for normalization. PCR primer pairs were designed to amplify only cDNA, had no reported off-target matches searching the human NCBI RefSeq database, and were within 80%–120% PCR efficiencies with single dissociation curves.

qPCR oligonucleotide primer pairs were as follows: *TPM1*-F, AGTCGAGCCCAAAAAGATGA; *TPM1*-R, TCGCTCTCAATGATGACCAG; *TPM2*-F, GGACAGAGGATGAGGTGGAA; *TPM2*-R, GCATCAGTGGCCTTCTTCTC; *TPM3*-F, CCTGCAAAAGCTGGAAGAAG; *TPM3*-R, TCTGCCTCTTCTGCAATGTG; *TPM4*-F, GGAGATGCAGCTCAAAGAGG; *TPM4*-R, CCTCCAGGATGACCAGCTTA; *GAPDH*-F, TATCGTGGAAGGACTCATGACC; *GAPDH*-R, TAGAGGCAGGGATGATGTTCTG.

### *TPM4* knockdown in human megakaryocytes

#### Short hairpin vector cloning.

Oligonucleotides were selected from the RNAi consortium database. Short hairpin or control nontarget sequences were annealed before cloning into the pLKO.1 lentiviral vector backbone (Addgene 10878) downstream of the human U6 promoter and checked for sequence integrity.

#### Viral particle production.

Replication-deficient lentiviral vector particles (LVPs) were produced by transient cotransfection of HEK 293T/17 cells (ATCC CRL-11268) with pLKO.1–short hairpin constructs along with the psPAX2 and pMD2.G helper plasmids (Addgene 12260, 12259) using TurboFect transfection reagent (Thermo Fisher Scientific). Crude supernatants containing LVPs were concentrated by PEG-based precipitation (LentiX-concentrator, Clontech) and functional titers determined by qPCR measurement of provirus copy number in genomic DNA of transduced HCT116 cells (ATCC CCL-247).

#### Oligonucleotides used to construct short hairpin vectors.

The following oligonucleotides were used to construct short hairpin vectors: TPM4_shA forward, CCGGGGAAGAGGCTGACCGCAAATACTCGAGTATTTGCGGTCAGCCTCTTCCTTTTTG; TPM4_shA reverse, AATTCAAAAAGGAAGAGGCTGACCGCAAATACTCGAGTATTTGCGGTCAGCCTCTTCC; TPM4_shB forward, CCGGTCAGACACTAAACGAACTTAACTCGAGTTAAGTTCGTTTAGTGTCTGATTTTTG; TPM4_shB reverse, AATTCAAAAATCAGACACTAAACGAACTTAACTCGAGTTAAGTTCGTTTAGTGTCTGA; TPM4_shE forward, CCGGCCCAGTATCTAGTCGTGGATACTCGAGTATCCACGACTAGATACTGGGTTTTTG; TPM4_shE reverse, AATTCAAAAACCCAGTATCTAGTCGTGGATACTCGAGTATCCACGACTAGATACTGGG.

### Statistical analysis of the effect of TPM4 level on proplatelet formation in human megakaryocytes

To analyze the effect of TPM4 RNA and protein level on proplatelet formation, we performed logistic regression. A mixed-effects model was fitted to the log odds of a cell being proplatelet forming. A coefficient was included representing the effect of an increase in RNA or protein levels on the log odds and was adjusted for cord effects using a random effect parameter.

### Preparation of washed platelets

#### Mouse.

Murine blood was obtained by cardiac puncture into 0.1 volume of Aster-Jandl anticoagulant (85 mM sodium citrate, 69 mM citric acid, and 20 mg/ml glucose, pH 4.6; ref. 54). Platelet-rich plasma was obtained by centrifugation at 125 *g* for 8 minutes, followed by centrifugation of the supernatant buffy coat at 125 *g* for 8 minutes. Platelets were washed by 2 sequential centrifugations at 860 *g* for 5 minutes in 140 mM NaCl, 5 mM KCl, 12 mM trisodium citrate, 10 mM glucose, and 12.5 mM sucrose, pH 6.0. The platelet pellet was resuspended in Tyrode-HEPES buffer (134 mM NaCl, 0.34 mM Na_2_HPO_4_, 2.9 mM KCl, 12 mM NaHCO_3_, 5 mM HEPES, pH 7.4) containing 5 mM glucose, 0.35% BSA, and 1 mM CaCl_2_.

#### Human.

Platelet-rich plasma was obtained from citrated human whole blood by centrifugation at either 50 *g* for 10 minutes (patients) or 150 *g* for 20 minutes (controls). Platelets were washed by centrifugation at 1,000 *g* for 10 minutes followed by resuspension in Tyrode-HEPES buffer containing 5 mM glucose before a final spin at 1,000 *g* for 10 minutes.

### Analysis of platelet activation and surface receptor expression by flow cytometry

#### Mouse.

Washed platelets were adjusted to a concentration of 5 × 10^4^ per microliter and activated with the indicated agonists for 15 minutes at room temperature in the presence of JON/A–PE and anti–P-selectin–FITC antibodies (diluted according to the manufacturers’ instructions). Samples were diluted with PBS and analyzed on a FACSCalibur. To assess platelet receptor expression, anticoagulated whole blood was diluted 1:20 using Tyrode-HEPES buffer and stained with platelet-specific antibodies or negative controls for 20 minutes at room temperature. Samples were diluted with Tyrode-HEPES buffer and directly acquired by flow cytometry.

#### Human.

Citrated whole blood was incubated for 5 minutes with hirudin and aspirin, before being added to tubes containing HEPES-buffered saline, FITC–rabbit anti-fibrinogen antibody (Dako, F0111), and PE–mouse anti–P-selectin (IBGRL, clone Thromb6) or relevant negative controls (P-selectin isotype, IBGRL, clone 9E10). Platelets were agonized with a final concentration of 0.0005 μM ADP, 0.3 μg/ml CRP-XL, and 0.8 μM TRAP-6 or left untreated. Apyrase was added to platelets agonized with CRP-XL and TRAP-6. Flow cytometry was performed on an FC-500 (Beckman Coulter), and platelets were identified by light scatter. Results were recorded as the percentage of platelets positive for the relevant activation marker.

### Platelet spreading

Coverslips were coated with 100 μg/ml human fibrinogen and blocked with PBS 1% BSA. After rinsing with Tyrode-HEPES buffer, washed mouse platelets (100 μl with 0.03 × 10^6^ platelets per microliter) were activated with 0.005 U/ml thrombin and incubated at room temperature for the indicated time periods. After fixation with 4% paraformaldehyde in PBS for 10 minutes, the coverslips were thoroughly rinsed with PBS, and platelets were visualized with a Deltavision Elite microscope equipped with CoolSnap2 CCD Detector (×100/1.4 oil immersion objective). Images were analyzed using ImageJ software.

### Platelet turnover studies in mice

Mice were injected i.v. with 0.15 μg/g body weight of X-488 (Emfret), a rat-derived IgG against the platelet glycoprotein Ibβ receptor, and platelet lifespan was measured as previously described (54).

### Tail bleeding assay

Mice were anesthetized using ketamine/xylazine. The tail was transected at 2 mm from the tip and immediately immersed in 37°C saline. The bleeding time was determined as the time from the tail transection to the cessation of blood flow or stopped after 10 minutes.

### Statistics

Data distribution was analyzed using the Shapiro-Wilk test. Statistical significance between 2 experimental groups was analyzed using an unpaired 2-tailed Student’s *t* test or a Mann-Whitney test. When more than 2 experimental groups were compared, data were analyzed using 1-way ANOVA. When ANOVA indicated that a significant difference was present, individual differences were explored with the 2-tailed unpaired Student’s *t* test or Mann-Whitney test using Bonferroni correction for multiple comparisons (Prism 7, GraphPad Software). *P* values less than 0.05 were considered as statistically significant; *P < 0.05, **P < 0.01, ***P < 0.001 or as otherwise stated. Data are presented as mean ± SD.

### Study approval

#### Patients.

The patients were enrolled to the BRIDGE-BPD study (UK REC10/H0304/66) and to the NIHR BioResource–Rare Diseases study (UK REC 13/EE/0325) after providing informed written consent. The control volunteers for functional work were enrolled to the Genes and Platelets study (UK REC10/H0304/65) after providing informed consent. For genetic analysis, control groups comprised other patients with bleeding or platelet disorders (BPD) of unknown genetic basis or with unrelated rare disorders enrolled to the NIHR BioResource–Rare Diseases study (UK REC 13/EE/0325).

#### Animal experiments.

All animal experiments complied with the regulatory standards of, and were approved by, the Walter and Eliza Hall Institute and the University of New South Wales Animal Ethics Committees.

## Author contributions

IP designed and performed experiments on mouse megakaryocytes and platelets, analyzed and interpreted data, and wrote the paper. JW designed and performed experiments on human megakaryocytes and platelets, analyzed and interpreted data, and wrote the paper. SC designed and performed experiments on mouse megakaryocytes and platelets and analyzed and interpreted data. VK designed and performed experiments on human megakaryocytes and platelets and analyzed data. NF designed and performed experiments on human megakaryocytes and platelets and analyzed data. JBB designed experiments for immunofluorescence stainings on human megakaryocytes and thrombus formation under flow and analyzed data. KA designed and performed experiments on mouse megakaryocytes and platelets and analyzed data. CL contributed to experiments on human megakaryocytes. RML contributed to animal experiments. GS interpreted results on the detection of tropomyosin isoforms. WSA and DJH contributed to the mouse ENU mutagenesis screen. WJA compared platelet parameters with distributions derived from INTERVAL data. KD performed functional analysis of human platelets. PN interpreted results from electron microscopy on human platelets. SKW and ADM recruited pedigree 1 and collected clinical phenotype data. SGO and PWC recruited pedigree 2 and collected clinical phenotype data. NIHR BioResource is a National Institute for Health Research research infrastructure that is responsible for the whole genome sequencing and phenotyping of 10,000 patients with rare diseases of unknown molecular etiology. FD, LMI, NSB, MH, and CAL generated and characterized the *Tpm4.2* knockout mouse. ECH conceived and supervised the generation and characterization of the *Tpm4.2* knockout mouse. WHO directs the Bleeding and Platelet Disorders project of the NIHR BioResource–Rare Diseases. PWG contributed to the design and interpretation of experiments on the detection and function of tropomyosins. ET analyzed and interpreted human genetic, RNA, and proteomic data. MRT conceived the study in humans, designed experiments, interpreted data, and wrote the paper. BTK conceived the study, designed experiments, interpreted data, and wrote the paper.

## Supplementary Material

Supplemental data

Supplemental Video 1

Supplemental Video 2

## Figures and Tables

**Table 3:**
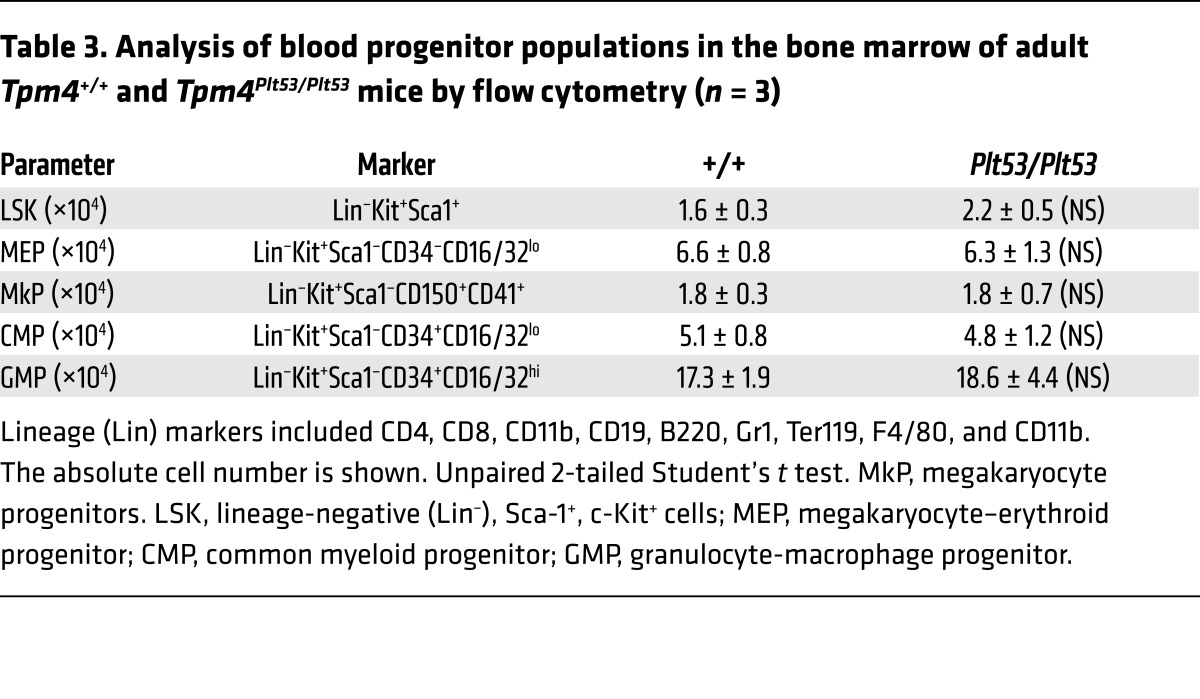
Analysis of blood progenitor populations in the bone marrow of adult *Tpm4^+/+^* and *Tpm4^Plt53/Plt53^* mice by flow cytometry (*n* = 3)

**Table 2:**
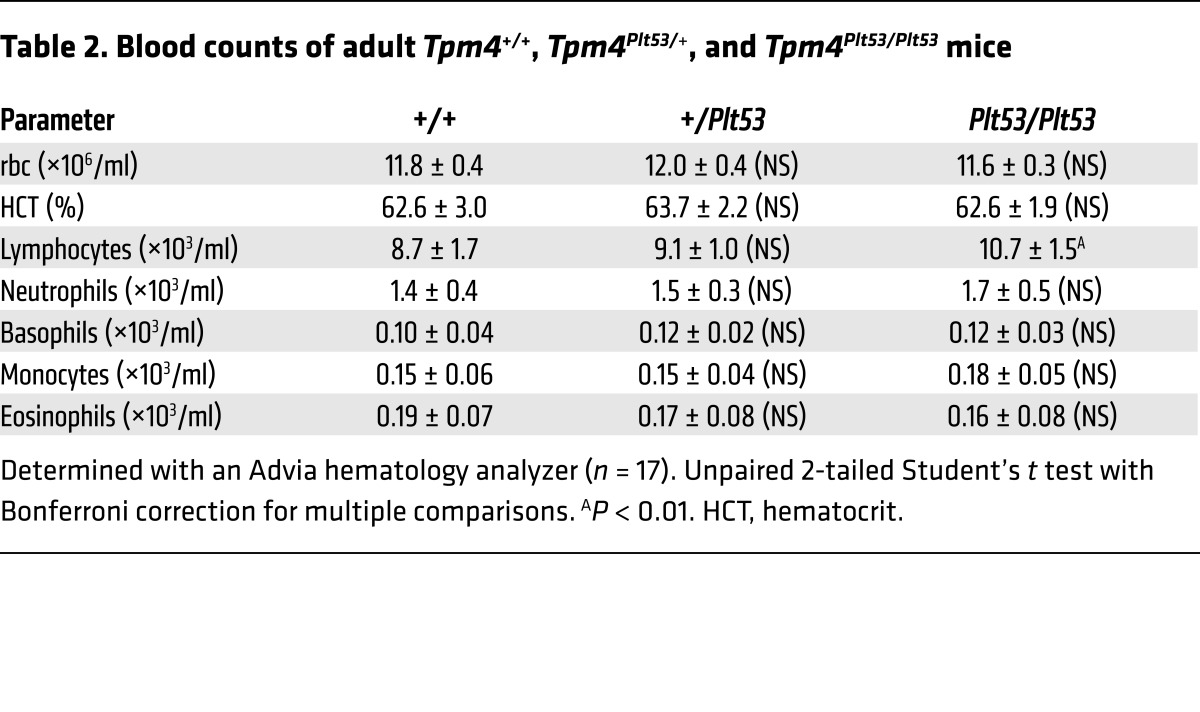
Blood counts of adult *Tpm4^+/+^*, *Tpm4*^Plt53/+^, and *Tpm4^Plt53/Plt53^* mice

**Table 1:**
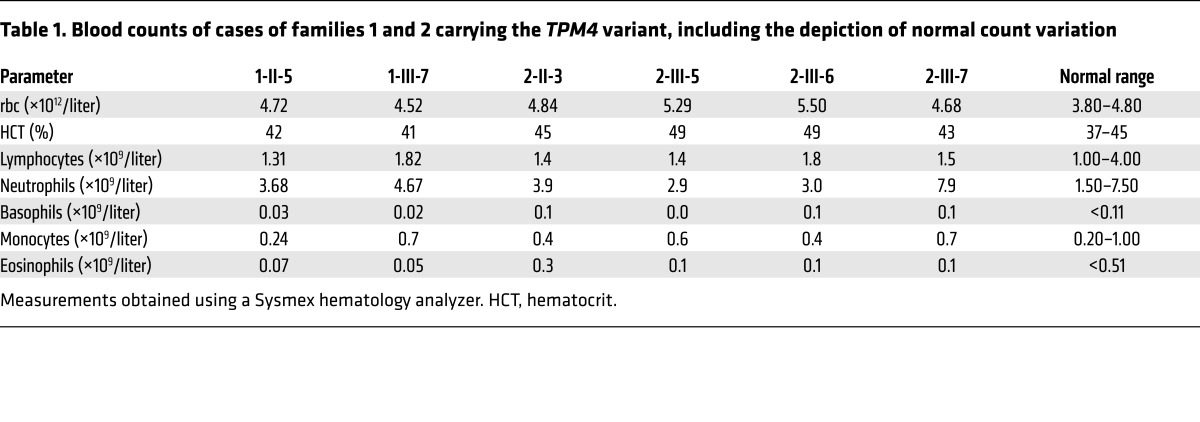
Blood counts of cases of families 1 and 2 carrying the *TPM4* variant, including the depiction of normal count variation
