# Supplementary material for: Mutations in tropomyosin 4 underlie a rare form of human macrothrombocytopenia
Source: J Clin Invest. 2017 Jan 30;127(3):814–29. doi: 10.1172/JCI86154 (PMC5330761; doi:10.1172/JCI86154)
Supplement: Supplemental data [file jci-127-86154-s001.pdf]

SUPPLEMENTAL DATA

Clinical history

The propositus of Pedigree 1's bleeding symptoms comprised prolonged bleeding after a previous mastectomy and axillary node clearance procedure for breast carcinoma that required blood transfusion. There was prolonged bleeding after extraction of one wisdom tooth, but no other pathological bleeding symptoms. Uterine dilatation and curettage, joint injection, three other wisdom tooth extractions, mole removal and a spontaneous vaginal delivery were not complicated by excessive bleeding.

The hysterectomy of the propositus of Pedigree 2 was complicated by post-operative bleeding for which she received a blood transfusion. Following two dental extractions, she had prolonged bleeding, which necessitated suturing to achieve haemostasis. She also reported intermittent heavy blood loss for up to 8 weeks following two first trimester miscarriages and after her first delivery. However, she did not require iron therapy or blood products. She did undergo a laparoscopic cholecystectomy, minor ophthalmic surgery and two spontaneous vaginal deliveries with no excessive bleeding.

Case 2-III-7 experienced bleeding for 3 days following a molar extraction, which eventually ceased with no intervention. She also suffered with longstanding menorrhagia and bled following a spontaneous vaginal delivery requiring iron replacement therapy.

Supplemental Methods

Materials. ENU, ADP, thrombin, Dimethyl sulfoxide (DMSO), propidium iodide (Sigma-Aldrich) and CVX (Enzo Life Sciences Inc.) were purchased. The enhanced chemiluminescence system was from Merck Millipore, the protease inhibitor cocktail, Complete, was from Roche. Rhodamine phalloidine and 4%-12% Bis-Tris gels (NuPAGE) were from Invitrogen Life technologies, and calibration beads 3.5–4.0 µm were from Spherotech Inc. X488 was obtained from Emfret Analytics. Anesthetic drugs xylazine (Rompun) and ketamine (Imalgene 1000) were from Bayer and Mérial Inc., respectively. Antibodies used for immunoblotting and immunofluorescence microscopy included: Rabbit anti-TPM4 exon 9d (#AB544) and mouse anti-TPM1/2 (15D12.2) from EMD Millipore; mouse anti-NMM-IIa (#ab55456), rabbit anti-ROCK (#ab71598) and

rabbit anti-TPM4 (EPR13317) from Abcam, rabbit anti-P-cofilin (Ser3, #3311) and rabbit anti-P-NMM-IIa (Ser1943, #5026) from Cell Signalling, mouse anti-RhoA (#ARH03) and rabbit anti-cofilin (#ACFL02) (Cytoskeleton), rabbit anti-Actinin1 (#HPA006035) from Sigma, rabbit anti-Filamin A (#PA5-29598 Thermo Scientific), and anti-actin-HRP (#sc-47778 Santa Cruz Biotechnology). Mouse anti-TPM4 exon 1b (clone #44), anti-TPM4 WD4/9d and anti-TPM3 (2G10.2) antibodies were generated as described (1). Antibodies used for flow cytometry to determine platelet activation and platelet surface receptor expression: Fluorescently conjugated GPIbα (Xia.G5), GPIX (Xia. B4), GPVI (JAQ1) antibodies, as well as the antibody directed against active integrin GPIIbIIIa/αIIbβ3 (JON/A) were obtained from Emfret Analytics.

Generation of BM chimeras. Lethally irradiated adult CD45.1 congenic Balb/c mice were transplanted with whole bone marrow of $Tpm4^{Plt53/Plt53}$ or $Tpm4^{+/+}$ mice (4×10^6 cells per recipient). Seven weeks after transplant, blood cell counts were measured.

Platelet transmission electron microscopy (TEM). *Human:* Human platelets were incubated for 20 minutes at 37°C before fixation with 0.2% glutaraldehyde. After incubation for 1hr at room temperature, cells were washed twice with saline solution before overnight fixation at 4ºC with 0.2% glutaraldehyde in 0.1 M sodium cacodylate buffer. This was followed by immersion in 2% formaldehyde (made from paraformaldehyde) and 2% vacuum distilled glutaraldehyde, containing 2 mM $CaCl_2$ in 0.05 M sodium cacodylate buffer pH 7.4 at 4ºC for 8 hours. Platelets were washed 5 times for 3 minutes in cold cacodylate buffer containing 2 mM $CaCl_2$ before incubation for 18 hours at 4ºC in 1% osmium ferricyanide followed by 5 washes with deionized water. Samples were then incubated for 30 minutes at room temperature in 1% thiocarbohydrazide followed by 5 washes with deionized water. Samples were incubated in 1% uranyl acetate in 0.05 M maleate buffer pH 5.5 for 48 hours at 4ºC before washing 5 times for 3 minutes in deionized water at room temperature. Platelets were dehydrated through 2 times each of 50%, 70%, 90% and 100% ethanol, followed by twice each in series of dry ethanol, dry acetone and dry acetonitrile, before being infiltrated with Quetol 651 epoxy resin over 5 days. The resin was cured for 48 hours at 65ºC before thin sections were prepared with a Leica Ultracut S, mounted on 200 mesh copper grids and

viewed with a Tecnia G2 operated at 200kV. *Mouse:* For visualization of the ultrastructure of mouse platelets, washed platelets were fixed with 2.5% glutaraldehyde in 0.1 M cacodylate buffer (pH 7.2). Samples were processed for TEM as described previously (2).

Megakaryocyte culture. *Human:* Cord blood was obtained after informed consent under a protocol approved by the National Research Ethics Service (07/MRE05/44). CD34-positive cells (>90%) isolated by magnetic cell sorting (Miltenyi Biotec), were seeded at $1x10^5$ cells/ml in CellGro SCGM medium (CellGenix) with 100 ng/ml human TPO (CellGenix) and 10 ng/ml IL-1b (CellGenix) and incubated for 10 days. On day 3 of culture, cells were transduced with lentivirus at 50 TU/cell before puromycin selection was added (day 6). CD41/CD42 expression was evaluated on day 10 before lysates were prepared for RNA and protein expression analysis. Flow cytometry was performed on a CyAn ADP 9 color (Beckman Coulter) using the following antibodies CD34PE (clone 581; Beckman Coulter), CD41aAPC (clone HIP8; BD Pharmingen), CD42aPE (clone ALMA.16; BD Pharmingen). For analysis of proplatelet formation, megakaryocyte maturity was verified by CD41/42 staining on day 8 of culture before cells were seeded at $7.5x10^4$ cells per well on 12-well chamber slides (Ibidi) pre-coated with fibrinogen (200 μg/ml, Enzyme Research Laboratories). After 48 hours cells were fixed with 1% formaldehyde and stained for TPM4 (rabbit polyclonal, #AB5449, Millipore), β-tubulin (#32-2588, Molecular Probes) and actin (phalloidin #R415, Molecular Probes), nuclei were counter-stained with DAPI (Fluoroshield, Sigma) and visualized and quantified using confocal microscopy (Leica SP5). *Mouse:* Fetal livers were removed at embryonic day (E) 13.5 and transferred into Dulbecco's modified Eagle's (DME) medium (high glucose version) with 10% fetal calf serum (FCS) (Gibco). Single cell suspensions were cultured for 3 days at 5×10^5 cells mL^{-1} in serum-free medium (SFM) (3) supplemented with 100 ng mL^{-1} murine thrombopoietin (WEHI) at 37°C, 5% CO_2, and mature megakaryocytes purified using a BSA gradient as described (4). On day 4, the percentage of proplatelet-forming megakaryocytes was determined using a light microscope. *Time lapse video microscopy of proplatelet formation*: Day 3 fetal liver megakaryocytes were enriched as detailed in the proplatelet formation assay and transferred to optically

transparent ibiTreat tissue culture treated sterile µ-slide 8-well plates (Ibidi). Experiments were performed using a Biostation IM-Q live cell time lapse imaging system (Nikon) equipped with a Nikon DS-Qil Digital Monochrome camera. Time-lapse images were acquired for 24 h using a 40×/0.8 objective with a 0.5x tube lense and processed using Image J software. *Immunofluorescence microscopy*: Day 4 megakaryocytes were spun onto glass slides, fixed using 4% paraformaldehyde, permeabilized with Triton X-100 and stained with the indicated antibodies. F-actin was visualized using phalloidin rhodamine and nuclei were stained with DAPI. Cells were analysed using a Zeiss LSM 780 confocal microscope equipped with ZEN software using a 40x/1.4 oil objective. The proplatelet tip diameter was measured using ImageJ software.

Determination of mouse megakaryocyte ploidy. Bone marrow was harvested from femora of 8- to 10-week-old mice into 1 mL of CATCH medium and megakaryocyte ploidy was studied by staining with CD41-FITC mAb and propidium iodide, as described (2).

Thrombus formation on collagen under flow. *Mouse*: Rectangular cover slips (24 × 60 mm) were coated with 200 µg/ml fibrillar type I collagen (Nycomed) and blocked with 1% BSA. Heparinized whole blood was labeled with a DyLight 488–conjugated anti-GPIX Ig derivative (0.2 µg/ml) and perfused over collagen-coated coverslips through a transparent flow chamber at a shear rate of 1,000 s–1 as previously described (5). Brightfield and fluorescence images were obtained from at least 7 different collagen-containing microscopic fields for each sample using a Zeiss Axiovert 200 inverted microscope (×40 objective; Carl Zeiss) and analysed off-line using Metavue software (Visitron). *Human:* Heparinised blood was pumped through a perfusion chamber containing collagen. Briefly, cover slips were locally coated with Horm collagen spots (50 µg/mL, Nycomed Linz Austria) and then used to assemble a flow chamber placed under a fluorescent microscope (EVOS system, Advanced Microscopy Group). The blood was then perfused for 3.5 min, followed by the perfusion of P-Selectin antibody (clone Thromb/6, NHSBT-Filton) for 2 min and a final wash step with Tyrode-Hepes buffer (supplemented with 3.3% BSA, 3.3% Glucose and 4% of 1M MgCl2) for 3

additional min. Solutions were perfused at 7.2 mL/h and 1,600 s^{-1}. Thrombi formation on collagen spots was recorded and analysed using ImageJ.

Measurement of platelet F-actin content. Washed mouse platelets ($0.02x10^6$/µL) were either left unstimulated or were stimulated with 1 U/mL thrombin for 2 minutes (37°C). After fixation with 0.55 volume of 10% paraformaldehyde in PBS (10 min) and permeabilization with 0.1 volume 1% Triton X-100 platelets were incubated with 10 µM phalloidin-FITC for 30 minutes and analysed on a FACSCalibur.

SDS-PAGE and Western blot analysis. *$Tpm4^{Plt53}$ mutant mice:* Mouse platelets and megakaryocytes were lysed in NP40 lysis buffer. Proteins were separated on 4-12% Bis-Tris gels (NuPAGE; Invitrogen) under reducing conditions, transferred onto Millipore PDVF membranes, and immunoblotted with various antibodies (see above), followed by incubation with secondary HRP-conjugated antibodies and ECL (GE Healthcare/Amersham, Pittsburgh, PA, USA). Densitometry analyses were carried out using Image J software. *Tpm4.2 knockout mice:* Protein was extracted from mouse tail tips in RIPA buffer and analysed by SDS-PAGE and Western blotting as described previously (1) Protein concentration was estimated using Precision Red (Cytoskeleton, Inc). Equal amounts of protein (25 µg) were resolved on a 12.5% SDS-PAGE gel before electro-transfer to PVDF membranes. Non-specific binding was blocked with blocking buffer 1% BSA in TBST (100 mM Tris-Cl, pH 7.5. 150 mM NaCl with 0.05% Tween 20). TPM4.2 was recognised using an affinity purified δ/9d rabbit polyclonal antibody (1:500 in blocking buffer) and secondary antibody donkey anti rabbit Ig-conjugated horseradish peroxidase (Jackson Immuno Research Laboratories, Suffolk, UK) (1:10,000 in blocking buffer). Primary antibody was incubated overnight followed by secondary for 2 h with 4 x 15 min washes. Blots were developed with the Western Lightning Chemiluminescence Reagent (Perkin Elmer Life Sciences; Boston, MA) and exposed to x-ray film. Equal protein loading was examined by staining the protein gel blots with 0.1% (w/v) Coomassie blue R350, 20% (v/v) methanol and 10% (v/v acetic acid). Western blots were scanned on a Biorad Gel doc imaging system and quantified using ImageJ software. *Human*: Platelets and megakaryocytes were lysed in RIPA buffer (150mM NaCl, 50mM Tris-HCl pH 8, 0.5% sodium deoxycholate, 0.1% SDS, 1% NP40)

containing protease inhibitors (complete EDTA-free protease inhibitor cocktail tablets Roche). Total cell lysates (5 μg of protein per lane) were separated using NuPage 4-12% Bis-Tris gel electrophoresis and transferred to polyvinylidenedifluoride (PVDF) membrane Immobilon-FL (Millipore) by electroblotting. Immunoblots were then probed with primary antibodies to GAPDH (14C10 Cell Signaling Technology) and β-actin (A5441 Sigma). TPM4.2 was recognised using an affinity purified δ/9d rabbit polyclonal antibody. Bands were visualized using ECL anti-rabbit IgG HRP or ECL anti-mouse IgG HRP (GE Healthcare) and ECL Clarity reagents (Bio-Rad), according to the manufacturer's instructions.

Supplemental References

1. Schevzov, G., Whittaker, S.P., Fath, T., Lin, J.J., and Gunning, P.W. 2011. Tropomyosin isoforms and reagents. *Bioarchitecture* 1:135-164.
2. Josefsson, E.C., James, C., Henley, K.J., Debrincat, M.A., Rogers, K.L., Dowling, M.R., White, M.J., Kruse, E.A., Lane, R.M., Ellis, S., et al. 2011. Megakaryocytes possess a functional intrinsic apoptosis pathway that must be restrained to survive and produce platelets. *J Exp Med* 208:2017-2031.
3. Norol, F., Vitrat, N., Cramer, E., Guichard, J., Burstein, S.A., Vainchenker, W., and Debili, N. 1998. Effects of cytokines on platelet production from blood and marrow CD34+ cells. *Blood* 91:830-843.
4. Shivdasani, R.A., and Schulze, H. 2005. Culture, expansion, and differentiation of murine megakaryocytes. *Curr Protoc Immunol* Chapter 22:Unit 22F 26.
5. Nieswandt, B., Brakebusch, C., Bergmeier, W., Schulte, V., Bouvard, D., Mokhtari-Nejad, R., Lindhout, T., Heemskerk, J.W., Zirngibl, H., and Fassler, R. 2001. Glycoprotein VI but not alpha2beta1 integrin is essential for platelet interaction with collagen. *EMBO J* 20:2120-2130.

Supplemental Table.

**Receptor**	+/+	***Plt53/+***	***Plt53/Plt53***
CD41	326 ± 23	380 ± 36 (NS)	447 ± 18 (***)
GPIb	54 ± 5	58 ± 4 (NS)	76 ± 7 (*)
GPIX	36 ± 12	38 ± 4 (NS)	45 ± 5 (*)
GPVI	103 ± 5	108 ± 14 (NS)	141 ± 5 (*)

Determination of levels of platelet surface receptors in $Tpm4^{+/+}$, $Tpm4^{Plt53/+}$ and $Tpm4^{Plt53/Plt53}$ platelets by flow cytometry (n=4). Values represent mean fluorescence intensities. Significances compared to the control are depicted. Unpaired 2-tailed students *t* test with Bonferroni correction for multiple comparisons. *$P < 0.05$; ***$P < 0.001$.

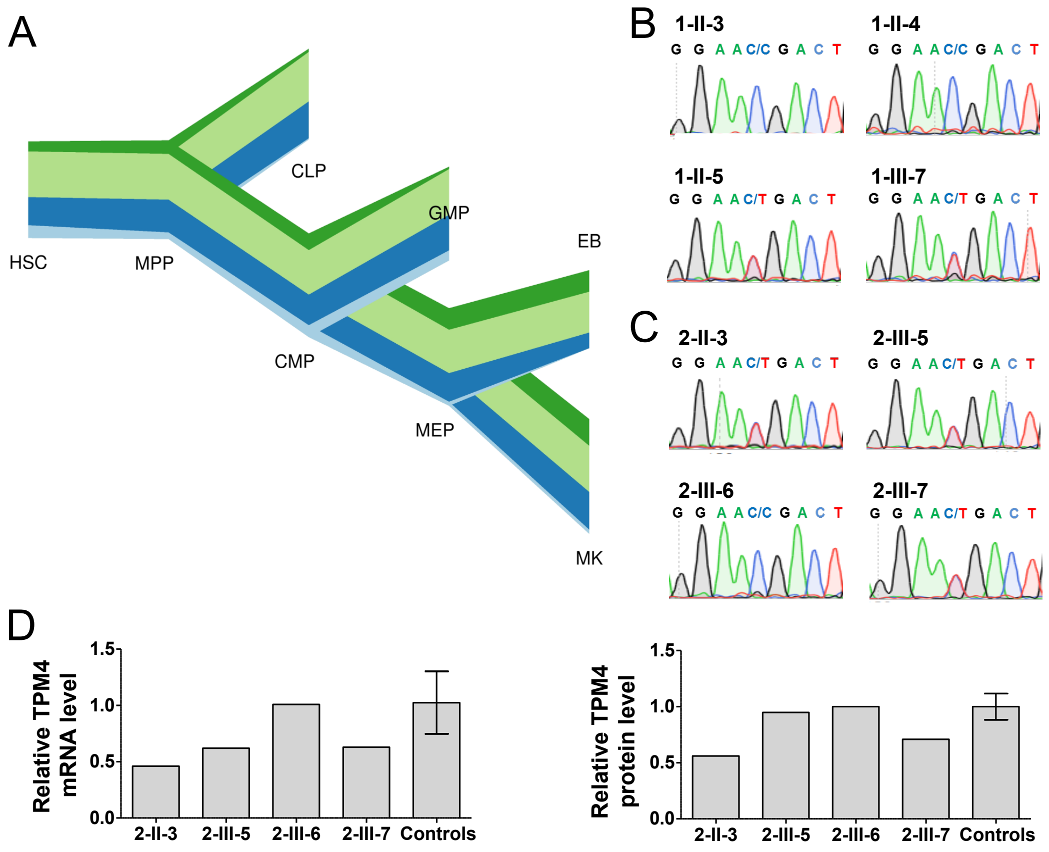

Supplemental Figure 1. (A) RNA sequencing data of human blood progenitor populations illustrating that human megakaryocytes express *TPM1*, *3* and *4,* and low levels of *TPM2* (1). **(B,C)** Sanger sequencing traces showing co-segregation of the observed variants with macrothrombocytopenia in **(B)** Pedigree 1 and **(C)** Pedigree 2. **(D)** Left: Platelet TPM4 RNA levels measured by RT-PCR using GAPDH as housekeeping gene showing TPM4 RNA levels are reduced in carriers of the mutation (2-II-3, 2-III-5 and 2-III-7) to 46-63% relative to controls but not in the unaffected family member (2-III-6). Graph depicts representative data from a total of n=3 measurements from 1 sample. Right: Platelet TPM4 protein levels measured by Western blot normalised to β-actin. Affected cases show TPM4 levels between 40-95% compared to controls.

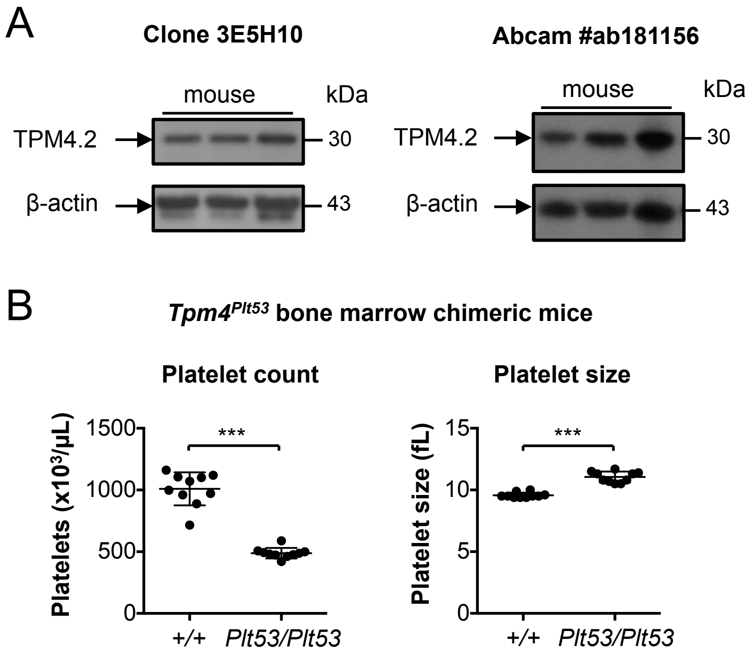

Supplemental Figure 2. (A) Western blot of mouse wild-type platelets using two different anti-TPM4 antibodies. Left: mouse monoclonal antibody clone 3E5H10 (2). Right: rabbit monoclonal antibody #ab181156 (Abcam). β-actin was used as loading control. **(B)** The *Plt53* phenotype is intrinsic to the haematopoietic system: macrothrombocytopenia in lethally irradiated wild-type mice transplanted with whole bone marrow of $Tpm4^{Plt53/Plt53}$ mice. Unpaired 2-tailed Student's *t* test. ***$P < 0.001$.

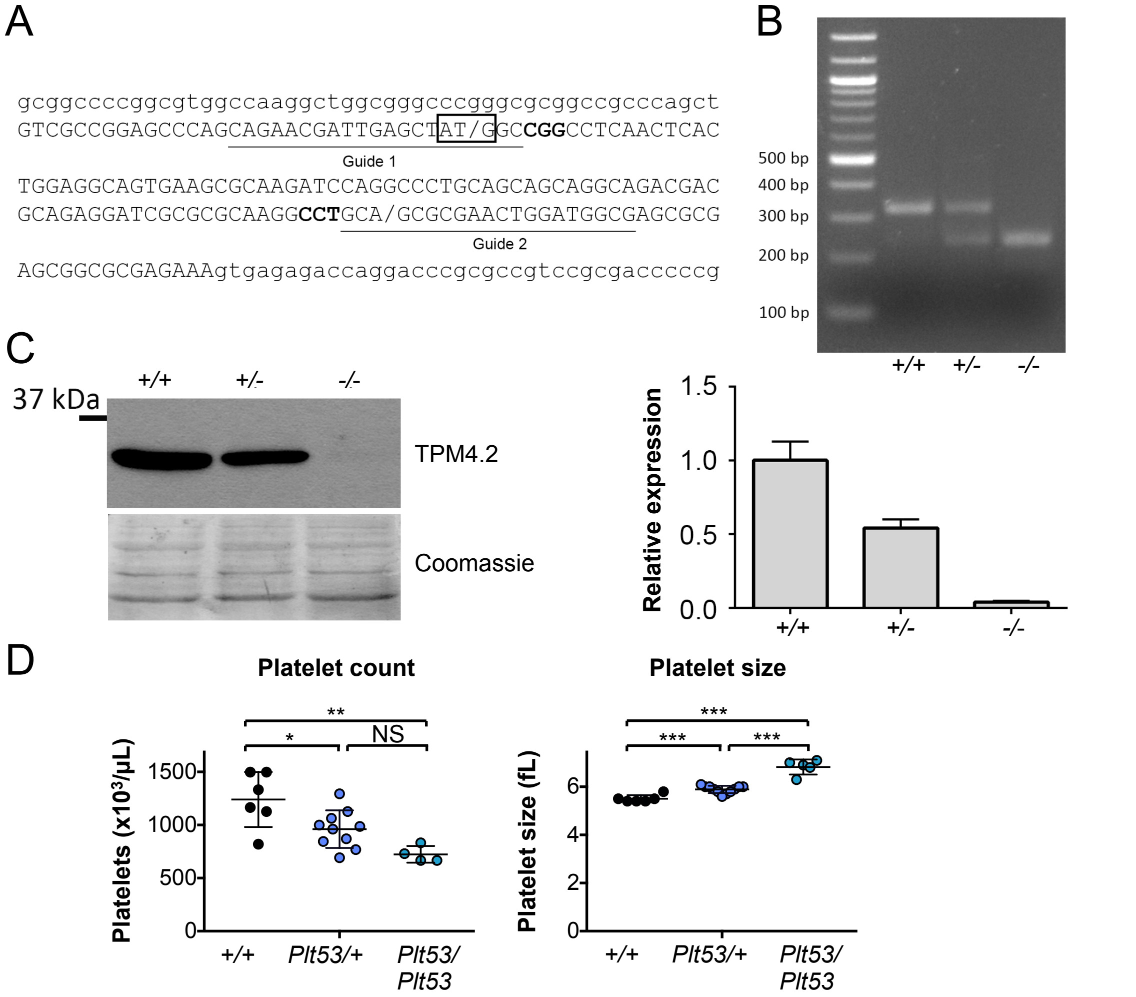

Supplemental Figure 3. (A-C) Generation of *Tpm4.2* knockout mice. **(A)** Genomic sequence of *Tpm4* exon 1b (3). Intronic sequence in lower case, exon in upper case. Guide sequences are underlined with the PAM sites in bold. Cut sites are indicated by / and the ATG start codon is boxed. **(B)** PCR based genotype determination of knockout (223 bp) and wild-type alleles (317 bp). **(C)** Left: Western blot of TPM4.2 protein expression in *Tpm4.2* wild-type (+/+), heterozygous (+/-), and homozygous (-/-) knockout mice. Equal protein loading is demonstrated by the Coomassie stained membrane. Right: Densitometry analysis. n=4 independent experiments. **(D)** Platelet count and size in $Tpm4^{Plt53}$ mutant mice (n=5-11) on a C57BL/6 background. Measurements were performed using a Sysmex haematology analyser. 1-way ANOVA followed by unpaired 2-tailed Student's *t* test with Bonferroni correction for multiple comparisons. *$P < 0.05$; **$P < 0.01$; ***$P < 0.001$.

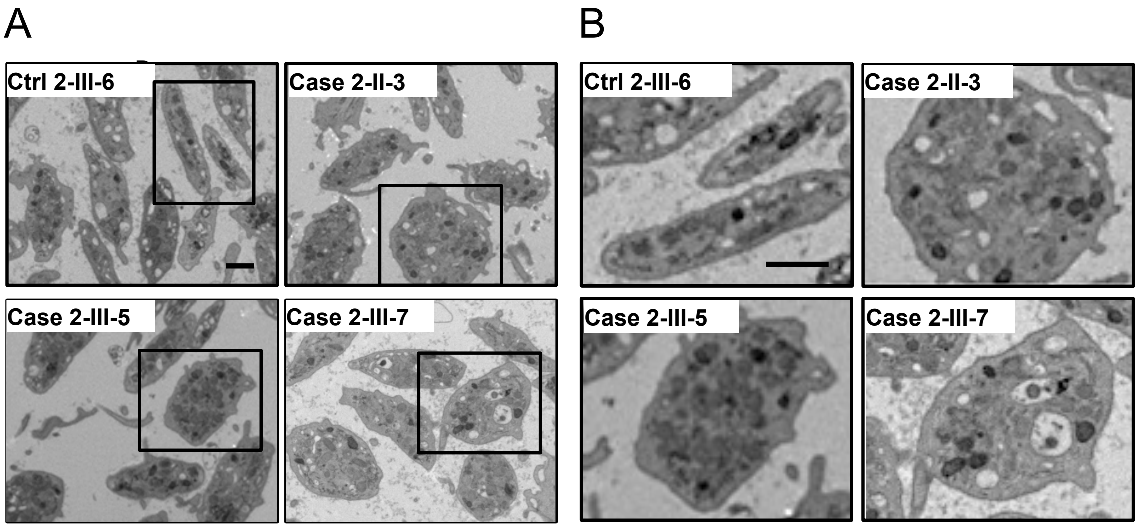

Supplemental Figure 4. (A,B) Ultrastructure of platelets from cases 2-II-3, 2-III-5 and 2-III-7 carrying the variant showing the presence of large platelets with numerous vacuoles indicating increased fragility, contrasting the normal discoid platelet appearance in 2-III-6 unaffected family member. **(A)** Overview. **(B)** Detail. Bars, 1 µm.

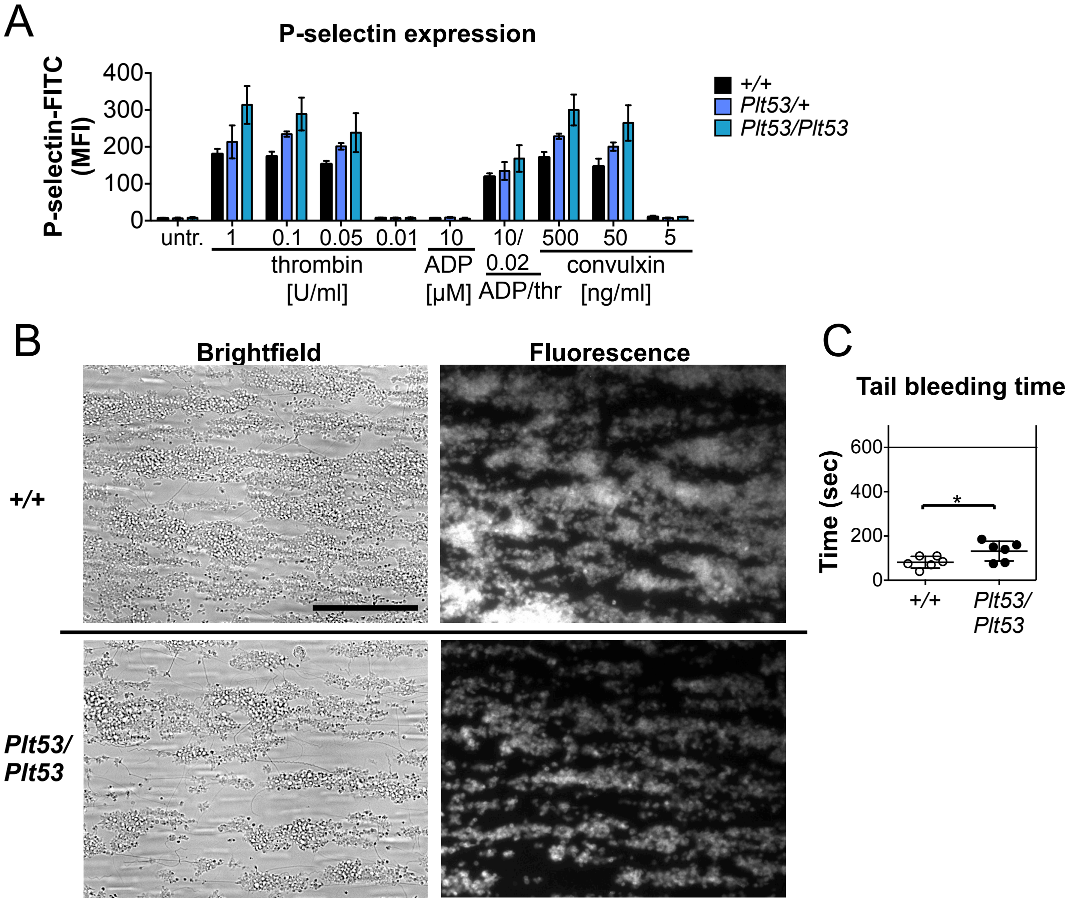

Supplemental Figure 5. (A) Flow cytometric measurement of α-granule release (P-selectin-FITC antibody) in *Tpm4*$^{+/+}$ (black), *Tpm*4$^{Plt53/+}$ (blue) and *Tpm4*$^{Plt53/Plt53}$ (light blue) platelets after activation with the depicted agonists (n=4, representative of three independent experiments). **(B)** Thrombus formation of whole, anti-coagulated blood from *Tpm4*$^{+/+}$ and *Tpm4*$^{Plt53/Plt53}$ mice on collagen under flow (shear rate 1000s-1). Representative bright field (left) and fluorescence images (right). Bar, 50 µm. **(C)** Tail bleeding times in bone marrow chimeric *Tpm4*$^{+/+}$ and *Tpm4*$^{Plt53/Plt53}$ mice. Each dot represents an individual. Unpaired 2-tailed Student's *t* test, *$P < 0.05$.

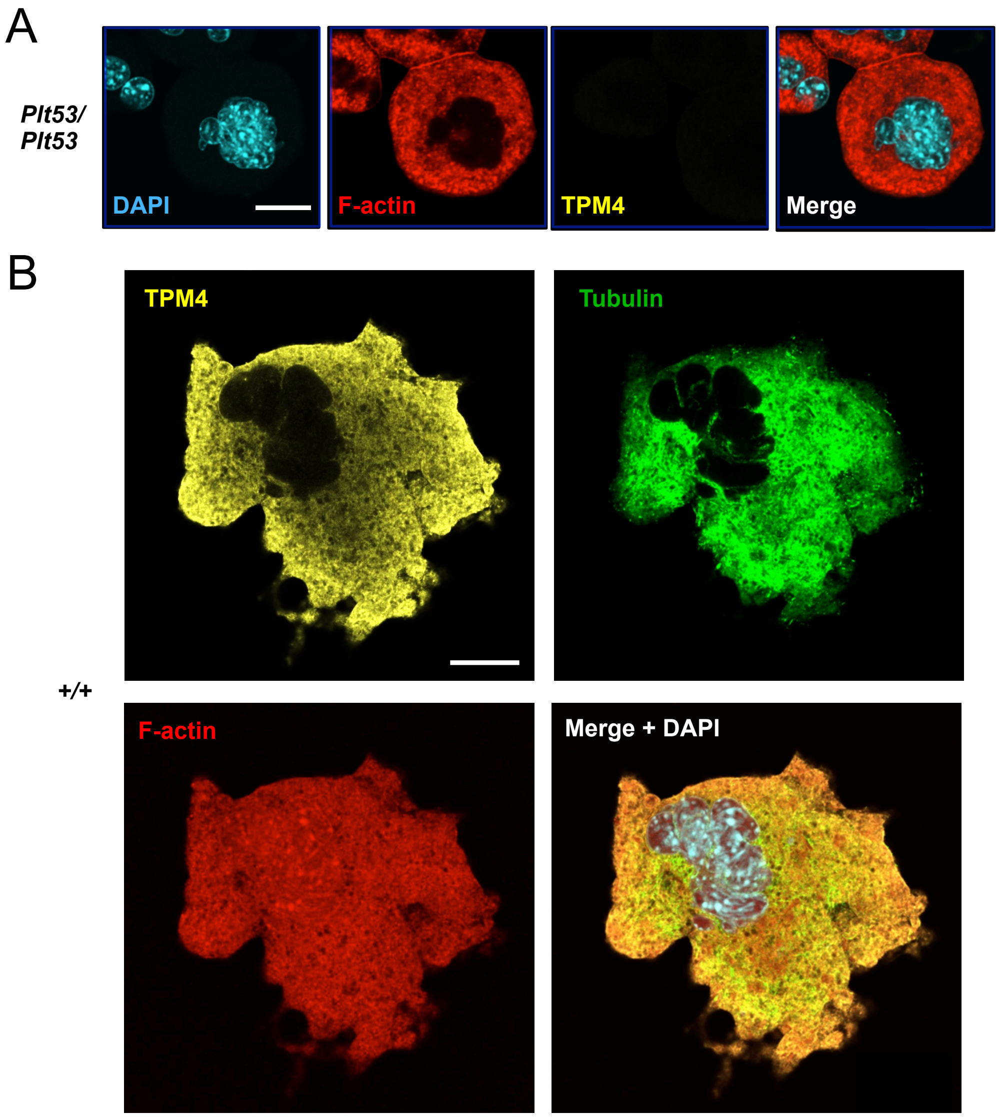

Supplemental Figure 6. (A) Investigation of TPM4 (yellow) and F-actin (red) localization in fetal liver cell-derived *Tpm4*$^{Plt53/Plt53}$ megakaryocytes by confocal immunofluorescence microscopy showing that TPM4 is not detectable. Bar, 20 µm. **(B)** Investigation of the localization of TPM4 (yellow), tubulin (green) and F-actin (red) in mature *Tpm4*$^{+/+}$ fetal liver cell-derived megakaryocytes at the start of proplatelet formation by confocal immunofluorescence microscopy. Bar, 20 µm.

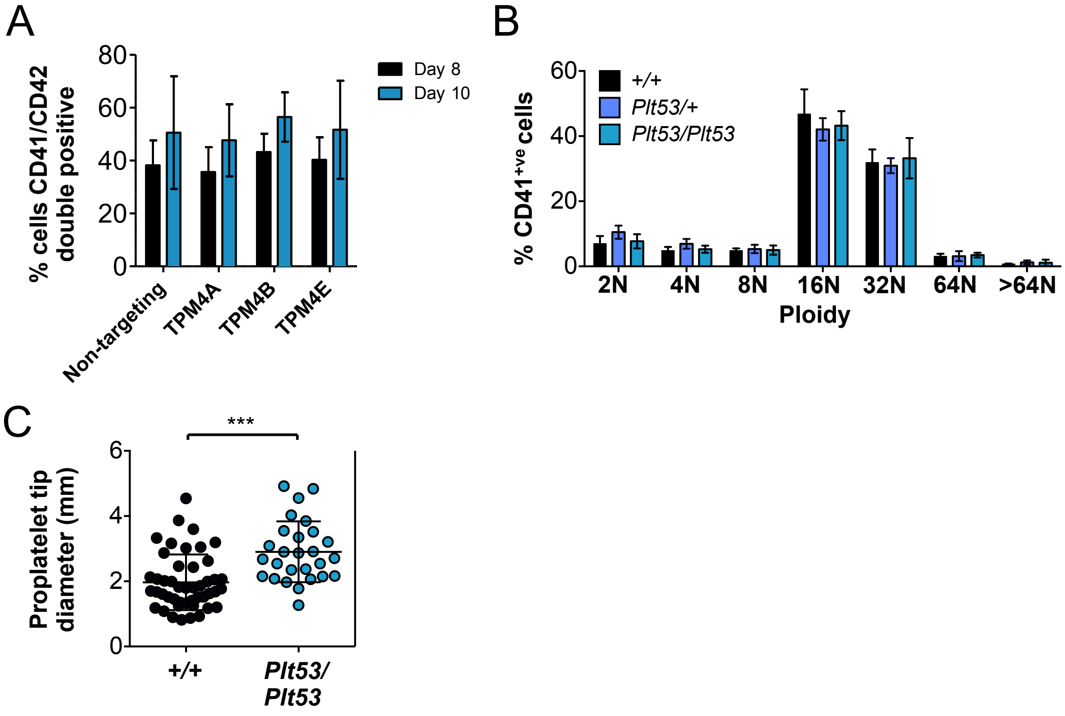

Supplemental Figure 7. (A) Knockdown of TPM4 expression by shRNA in human CD34+ derived megakaryocytes. Evaluation of megakaryocyte maturation at day 8 and day 10 of culture by CD41/CD42 surface staining is shown (n=5). **(B)** Normal ploidy of $Tpm4^{+/Plt53}$ and $Tpm4^{Plt53/Plt53}$ megakaryocytes. Whole bone marrow was incubated with propidium iodide and CD41-FITC antibody and ploidy of CD41-positive cells was determined by flow cytometry (n=5). **(C)** Examination of proplatelet tip size of cultured fetal liver derived $Tpm4^{+/+}$, and $Tpm4^{Plt53/Plt53}$ megakaryocytes. Each dot represents a proplatelet tip. Results are pooled from two independent experiments. Mann Whitney test, ***$P < 0.001$.

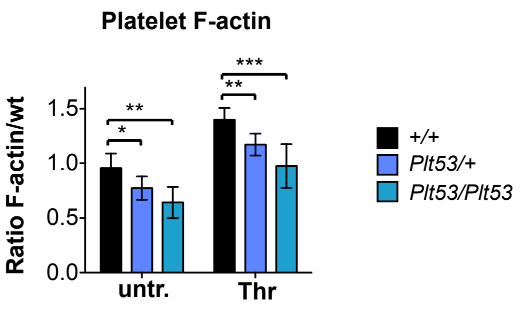

Supplemental Figure 8. Decreased F-actin content relative to the platelet size in non-stimulated and thrombin-stimulated *Tpm4*$^{Plt53/+}$ (blue) and *Tpm4*$^{Plt53/Plt53}$ (light blue) compared to *Tpm4*$^{+/+}$ (black) platelets (n=4). 1-way ANOVA followed by unpaired 2-tailed Student's *t* test with Bonferroni correction. *$P < 0.05$; **$P < 0.01$; ***$P < 0.001$.

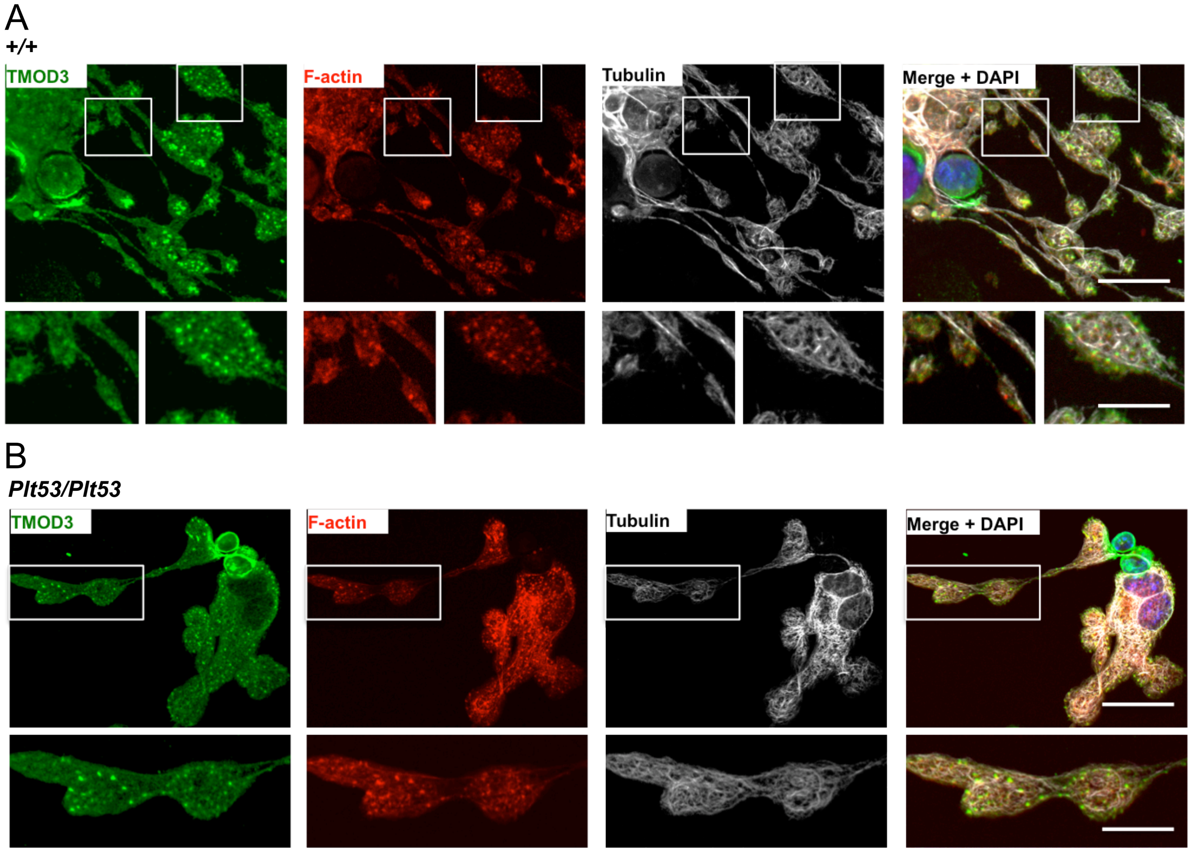

Supplemental Figure 9. Investigation of Tropomodulin 3 (TMOD3, green), F-actin (red) and Tubulin (grey) localisation in fetal liver cell-derived *Tpm4*$^{+/+}$ **(A)** and *Tpm4*$^{Plt53/Plt53}$ **(B)** megakaryocytes by confocal immunofluorescence microscopy. Overview: Bar, 20 µm. Detail: Bar, 10 µm.

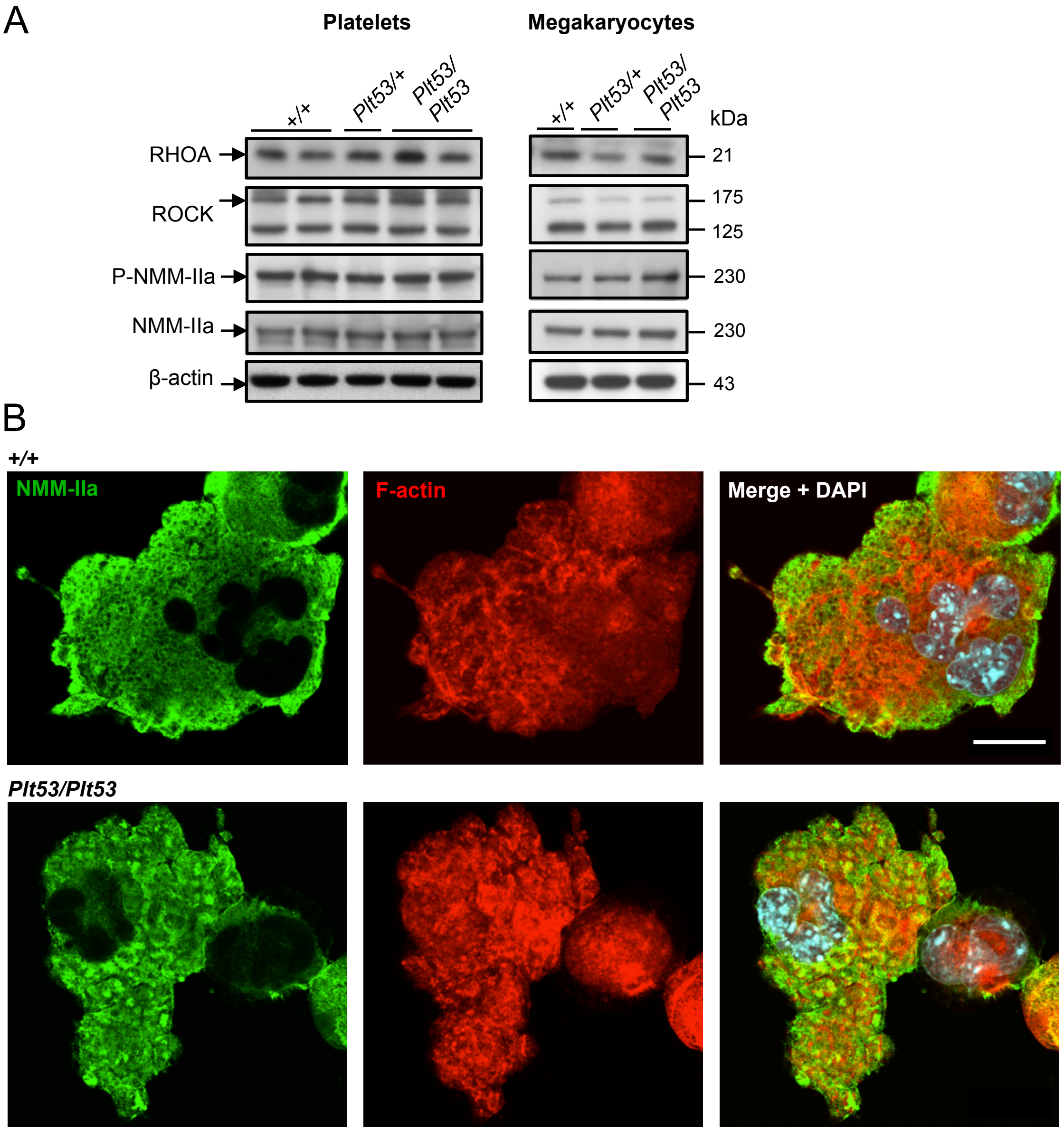

Supplemental Figure 10. (A) Investigation of potential cytoskeletal regulators affected in *Tpm4*$^{Plt53/+}$ and *Tpm4*$^{Plt53/Plt53}$ platelets (left) and fetal liver cell-derived megakaryocytes (right) by Western blot. Blots are representative of 2-3 individual experiments. β-actin was used as loading control. **(B)** Investigation of the localization of NMM-IIa (green) and F-actin (red) in *Tpm4*$^{+/+}$ and *Tpm4*$^{Plt53/Plt53}$ fetal liver cell-derived megakaryocytes by confocal immunofluorescence microscopy. Bar, 20 µm.
